# MicroRNA-21 is immunosuppressive and pro-metastatic via separate mechanisms

**DOI:** 10.1038/s41389-022-00413-7

**Published:** 2022-07-11

**Authors:** Lap Hing Chi, Ryan S. N. Cross, Richard P. Redvers, Melissa Davis, Soroor Hediyeh-zadeh, Suresh Mathivanan, Monisha Samuel, Erin C. Lucas, Kellie Mouchemore, Philip A. Gregory, Cameron N. Johnstone, Robin L. Anderson

**Affiliations:** 1 Olivia Newton-John Cancer Research Institute, HeidelbergVIC, Australia; 2 Immunology Division, The Walter and Eliza Hall Institute of Medical Research, Parkville, VIC Australia; 3 Bioinformatics Division, The Walter and Eliza Hall Institute of Medical Research, Parkville, VIC Australia; 4 Department of Biochemistry and Genetics, La Trobe Institute for Molecular Science, La Trobe University, Melbourne, VIC Australia; 5 Division of Immunology and Allergy, Department of Medicine, Karolinska Institute, Solna, Sweden; 6 School of Cancer Medicine, La Trobe University, Bundoora, VIC Australia; 7 Centre for Cancer Biology, University of South Australia, Adelaide, SA Australia; 8 Faculty of Health and Medical Sciences, The University of Adelaide, Adelaide, SA Australia; 9 Department of Clinical Pathology, University of Melbourne, Melbourne, VIC Australia; 10 Peter MacCallum Cancer Centre, Parkville, VIC Australia

**Keywords:** Breast cancer, siRNAs

## Abstract

MiR-21 was identified as a gene whose expression correlated with the extent of metastasis of murine mammary tumours. Since miR-21 is recognised as being associated with poor prognosis in cancer, we investigated its contribution to mammary tumour growth and metastasis in tumours with capacity for spontaneous metastasis. Unexpectedly, we found that suppression of miR-21 activity in highly metastatic tumours resulted in regression of primary tumour growth in immunocompetent mice but did not impede growth in immunocompromised mice. Analysis of the immune infiltrate of the primary tumours at the time when the tumours started to regress revealed an influx of both CD4^+^ and CD8^+^ activated T cells and a reduction in PD-L1^+^ infiltrating monocytes, providing an explanation for the observed tumour regression. Loss of anti-tumour immune suppression caused by decreased miR-21 activity was confirmed by transcriptomic analysis of primary tumours. This analysis also revealed reduced expression of genes associated with cell cycle progression upon loss of miR-21 activity. A second activity of miR-21 was the promotion of metastasis as shown by the loss of metastatic capacity of miR-21 knockdown tumours established in immunocompromised mice, despite no impact on primary tumour growth. A proteomic analysis of tumour cells with altered miR-21 activity revealed deregulation of proteins known to be associated with tumour progression. The development of therapies targeting miR-21, possibly via targeted delivery to tumour cells, could be an effective therapy to combat primary tumour growth and suppress the development of metastatic disease.

## Introduction

The development of distant metastatic disease is the major cause of death for patients with breast cancer. There has been a concerted effort over many years to identify genes that regulate the metastatic process with the aim of developing therapies capable of preventing or eliminating metastatic lesions. Many publications have reported protein-encoding genes that modulate the process and more recently, non-coding RNA genes have been implicated. Most of these are microRNA (miRNA) genes, including the oncogenic miRNAs, miR-10b [1], miR-155 [2], miR-373 [3] and others as reviewed recently [4]. In addition, tumour-suppressing miRNAs, such as the miR-200 family [5], the let-7 family [6], miR-335 [7] and miR-31 [8] have been reported. More recently, there has also been increasing evidence of long non-coding RNAs that regulate tumour progression [9–11].

Another oncogenic microRNA and the topic of this study, is miR-21 that has been reported to be highly expressed in many cancer types, compared to the corresponding normal tissue [12–15]. In addition, high levels of circulating cell-free miR-21 are a poor prognostic factor in some cancers [16, 17]. In breast cancer, several studies have reported poorer patient prognosis associated with either high levels of circulating miR-21 [18–20], or high tumour expression of miR-21 [21, 22]. One study showed that upregulation of miR-21 in the neoplastic cells of hormone receptor-positive cancers was associated with poor prognosis while elevated stromal levels of miR-21 were associated with poorer outcome for patients with triple negative breast cancer [23].

Increased tumour cell proliferation and invasion have been shown previously following elevation of miR-21 levels [24–26]. However, there are conflicting results for proliferation following knockdown of miR-21 in preclinical models [27, 28]. In breast cancer, a reduction in miR-21 levels can result in reduced primary tumour growth, for example in MCF-7 xenografts [24, 29], but this had little effect on MDA-MB-231 primary tumour growth [27]. Using experimental metastasis assays, it was shown that the knockdown of miR-21 reduced MDA-MB-231 lung metastasis [27] or bone colonization [30] following injection of tumour cells into the tail vein. Enhanced expression of miR-21 in the lungs of transgenic mice potentiated formation of non-small cell lung cancers driven by an activated K-Ras oncogene [31], while homozygous deletion of the miR-21 gene in mice reduced the incidence of lung tumours in the same K-Ras driven model [31]. Similarly, miR-21 genetic deletion increased the latency of mammary tumour formation in the MMTV-PyMT transgenic mouse model of breast cancer, and reduced both the number and size of metastases arising in the lungs [32]. Remarkably, miR-21 expression in the host stroma was required for successful engraftment of miR-21 wild-type PyMT tumours into the mouse mammary gland [32].

MiR-21 directly targets several tumour suppressor genes, thereby increasing the aggressiveness of tumour cells. Phosphatase and tensin homologue (*PTEN*) [25, 33] and genes involved in suppression of cell invasion and metastasis, including programmed cell death 4 (*PDCD4*) [33–35], maspin [27, 35], *TIMP3* [36], *RECK* [36], *SMAD7* [37, 38] and tropomyosin (*TPM1*) [39] were all reported to be down-regulated by miR-21. However, the repertoire of genes regulated by miR-21 varies among different tissues or cell types.

It is now well established that cancer cells communicate through exosomes and larger extracellular vesicles, which are known to contain proteins, lipids and nucleic acids that reflect the cell type from which they were derived [40]. Consequently, given the high levels of miR-21 in many cancer cells, miR-21 containing exosomes and extracellular vesicles in plasma are being explored as potential diagnostic and prognostic biomarkers of tumour presence, progression and response to therapy [41, 42]. Tumour-derived exosomes have also been implicated in the establishment of the premetastatic niche [43], with exosome-derived miR-21 shown to have an involvement in breast cancer metastasis to bone [44].

In this study, we demonstrate that suppression of tumour-derived miR-21 results in a profound inhibition of tumour growth in immunocompetent mice due to engagement of the adaptive immune response. In immune compromised mice, loss of tumour cell miR-21 has no impact on primary tumour growth but can still suppress tumour cell dissemination, presumably through a mechanism independent of the host immune system.

## Results

### MiR-21 is associated with aggressive properties of breast cancer

**Fig. 1:**
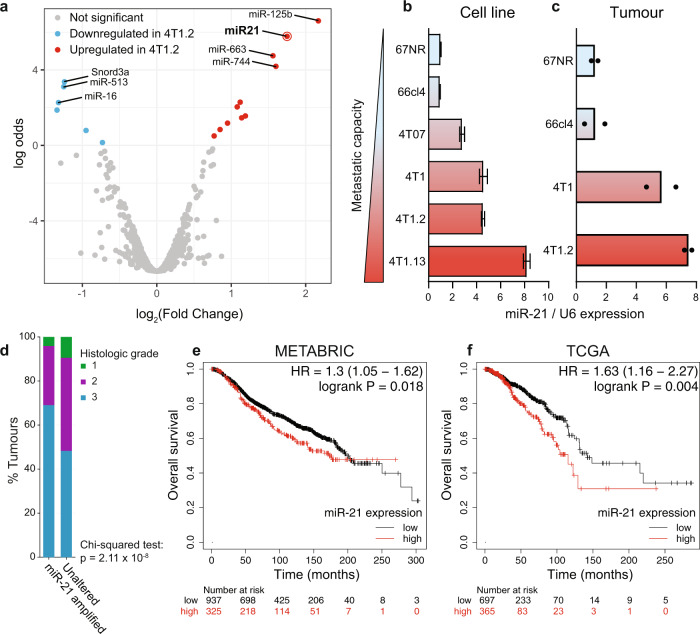
miR-21 is associated with metastatic capacity of breast cancer and predicts poor clinical outcome. **a** Volcano plot of differentially expressed miRNAs between non-metastatic 67NR and highly metastatic 4T1.2 mouse mammary tumour cells. **b**, **c** Expression of mature miR-21 levels in mouse mammary tumour cells (**b**) and in whole tumours (**c**) derived from the 4T1 model, with varying levels of metastatic capacity. MiR-21 expression was normalised to expression of Rnu6 small nuclear RNA (U6) and set to 1 in the non-metastatic 67NR line. Error bars in (**b**) represent mean ± SEM (*n* = 3). The mean and range (*n* = 2) is shown in (**c**). **d** The miR-21 gene at 17q23 is amplified in a subset of primary human breast cancers and is associated with higher tumour grade compared to non-amplified tumours (*p* = 2.11 ×10^−^^8^). **e**, **f** Higher levels of miR-21 expression in primary breast tumours are associated with poorer overall survival in the Metabric (*n* = 1262) and the TCGA (*n* = 1078) breast cancer patient datasets.

An analysis of differential miRNA expression between two isogenic mammary tumour lines, 67NR (non-metastatic) and 4T1.2 (highly-metastatic) [45, 46] revealed several differentially expressed miRNAs (Fig. 1a). MiRNAs highlighted in this screen included miR-125b, miR-663 and miR-744 that were up-regulated in 4T1.2 cells and miR-16, miR-513 and Snord3a, a small nucleolar RNA that were down-regulated. Of particular interest was the differential expression of miR-21 (Fig. 1a), confirmed by RT-qPCR in cells (Fig. 1b) and in primary tumours from variants of the 4T1 model (Fig. 1c). Similarly, miR-21 was up-regulated in mouse mammary tumour lines compared to NMuMG (immortalised mammary epithelial cells) (Fig. S1a). Analyses of published breast cancer datasets indicated that elevated miR-21 expression was associated with both higher grade tumours (Fig. 1d) and poorer patient survival (Fig. 1e, f).

### Modification of miR-21 levels in mammary tumour lines

**Fig. 2:**
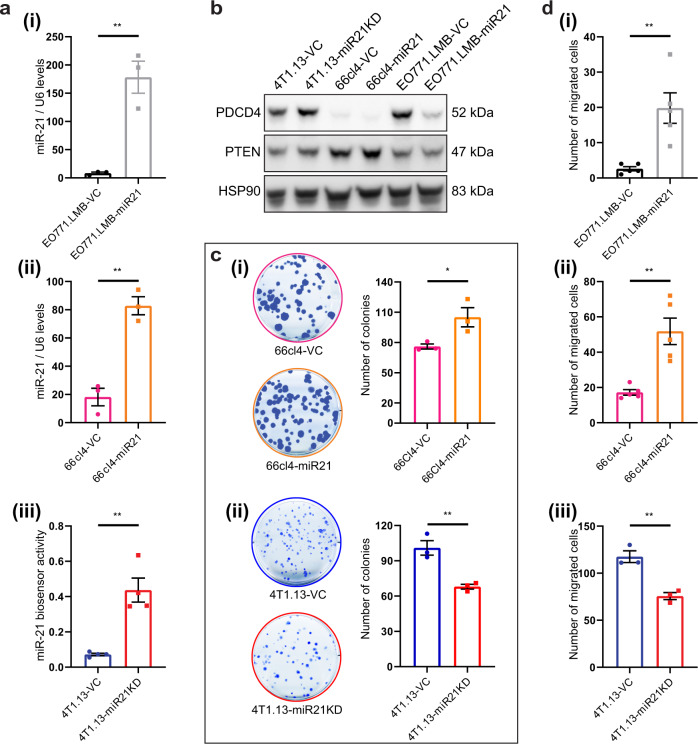
Effect of miR-21 on the phenotype of mammary cancer cells. **a** Confirmation of elevated expression of miR-21 in EO771.LMB and 66Cl4 tumour lines and suppression of miR-21 activity in 4T1.13 cells. For (i-ii), miR-21 transcript levels were normalised to expression of Rnu6 small nuclear RNA (U6). Mean ± SEM (*n* = 3). For (iii), reduced miR-21 activity was confirmed using a miR-21 specific luciferase-based reporter gene assay. Mean ± SEM (*n* = 4). **b** Western blot analysis of the miR-21 targets PDCD4 and PTEN in miR-21 modulated mouse mammary tumour lines. Heat shock protein 90 (HSP90) was used a loading control. **c** Extent of colony formation in 66cl4 and 4T1.13 cells after modulation of miR-21 activity. Mean ± SEM (*n* = 3). **d** Extent of chemotactic migration of EO771.LMB (i), 66cl4 (ii) and 4T1.13 (iii) cells after modulation of miR-21 activity. Mean ± SEM (*n* = 3–5). *p < 0.05; **p < 0.01.

To explore the role of miR-21 in tumour progression, exogenous miR-21 was stably expressed in two poorly metastatic lines, EO771.LMB and 66cl4 (Fig. 2a(i), (ii)) and mature miR-21 activity was stably repressed in the highly metastatic 4T1.13 line (Fig. 2a(iii)). Since the method used to reduce miR-21 activity involved sequestration of mature miR-21 RNA, the reduction in activity was validated using a miR-21-dependent reporter gene (Fig. 2a(iii)) and by expression analysis of known miR-21 target proteins, PTEN and PDCD4 (Fig. 2b). While protein levels of PTEN were not markedly altered by changes in miR-21 activity (Fig. 2b), PDCD4 responded to altered miR-21 in EO771.LMB and 4T1.13 cells, decreasing with higher miR-21 levels (Fig. 2b). Elevated miR-21 in EO771.LMB or 66cl4 cells reduced the activity of a miR-21 regulated 3′UTR reporter gene [47] (Fig. S1b(i), (ii)), demonstrating the appropriate functional activity of miR-21.

### Consequences of altered miR-21 levels on cellular phenotypes

To determine the influence of miR-21 on tumour cell intrinsic responses, we first measured their proliferative, migratory and invasive properties. In all three cell lines, in vitro proliferation was not substantially altered by modification of miR-21 activity (Fig. S1c), however, miR-21 promoted colony formation in 66cl4 cells (Fig. 2c(i)) with a corresponding loss of colony formation in the miR-21 suppressed 4T1.13 cells (Fig. 2c(ii)). While chemotactic migration was promoted by enforced miR-21 (Fig. 2d(i), (ii)), knockdown again had the opposite effect in 4T1.13 cells (Fig. 2d(iii)). However, there was no change in invasion through basement membrane (Fig. S1d).

### MiR-21 promotes tumour growth and metastasis

**Fig. 3:**
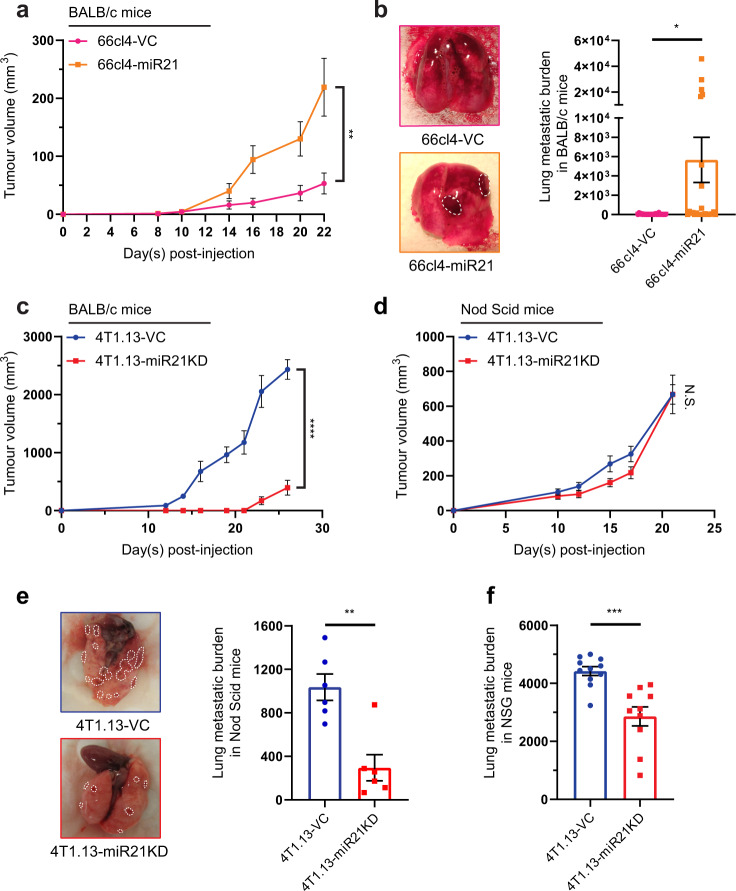
miR-21 promotes tumour growth and metastasis through immune-dependent and immune-independent mechanisms. **a** Effect of enhanced expression of miR-21 on the growth of 66cl4 tumours following inoculation of 1 × 10^5^ cells into the mammary fat pad of BALB/c mice. Mean ± SEM shown for each timepoint. *n* = 17 for the 66l4-VC group, *n* = 15 for the 66cl4-miR-21 group. **b** Spontaneous metastasis to lung following growth of 66cl4-VC or 66cl4-miR-21 mammary tumours (inoculation of 5 × 10^5^ cells) measured by qPCR of the reporter gene (mCherry) expressed in the tumour cells. Primary tumours were resected at the same size (~350 mm^3^). Mean ± SEM (*n* = 25/group). Representative images of lungs at the time of necropsy shown with dotted white lines delineating metastatic nodules. **c** Effect of inhibition of miR-21 activity on the growth of 4T1.13 mammary tumours following inoculation of 2 × 10^5^ cells into the mammary fat pad of BALB/c mice. Mean ± SEM (*n* = 8 for the 4T1.13-VC group and *n* = 9 for the 4T1.13-miR-21-KD group). **d** Effect of inhibition of miR-21 activity on the growth of 4T1.13 mammary tumours following inoculation of 2 × 10^5^ cells into the mammary fat pad of NOD-SCID mice. Mean ± SEM (*n* = 6/group). **e**, **f** Spontaneous metastasis of 4T1.13-VC and 4T1.13-miR-21-KD cells to lungs of NOD-SCID mice **e** (mean ± SEM, *n* = 6/group). and to lungs of NSG mice **f** (mean ± SEM, *n* = 10/group). For the NOD-SCID mice, representative images of lungs at the time of necropsy are shown with dotted white lines delineating metastatic nodules. *p < 0.05; **p < 0.01; ***p < 0.001; ****p < 0.0001.

Ectopic miR-21 expression in 66cl4 tumours enhanced both primary tumour growth rate (Fig. 3a) and spontaneous metastasis to lung in BALB/c mice (Fig. 3b). Intriguingly, suppression of miR-21 activity in 4T1.13 cells resulted in highly impaired primary tumour growth in BALB/c mice (Fig. 3c), even when the cell inoculum was increased 5-fold to 1 × 10^6^ cells (Fig. S2a). To determine if the inability to form tumours was cell intrinsic or whether changes in immune responsiveness caused their failure, we assessed tumour growth in immunocompromised mice with differing levels of immune impairment. In NOD-*Scid* (Fig. 3d), BALB/c nu/nu (nude) (Fig. S2b) and NOD-*Scid*-gamma (NSG) mice (Fig. S2c), miR-21-KD tumours grew at the same rate as the vector control tumours, indicating that the reduced growth of miR-21-KD tumours was cell extrinsic and likely to be immune-mediated. Of note, even with primary tumour growth restored, there remained a significant inhibition of spontaneous metastasis to lung caused by the loss of miR-21 activity (Fig. 3e, f).

In summary, in vitro and in vivo analyses using both immunocompetent and immunocompromised mice have shown that miR-21 influences breast cancer progression by two separate mechanisms. First, reduced miR-21 activity results in an apparent change in immune responsiveness that otherwise permits tumour growth in immunocompetent mice. Second, miR-21 has an additional activity, independent of immune regulation and potentially tumour cell intrinsic, that drives spontaneous metastasis to distant organs by enhancing cell motility and colony forming potential.

### Regulation of the anti-tumour immune response by miR-21

**Fig. 4:**
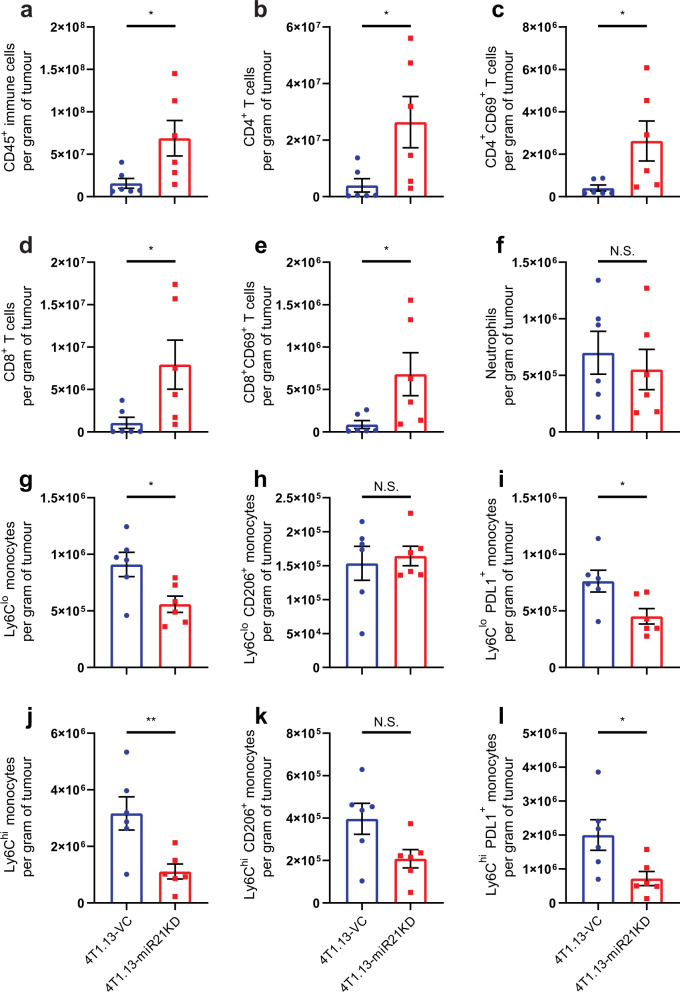
Effect of miR-21 suppression on the immune profile of 4T1.13 primary tumours. BALB/c mice bearing 4T1.13-VC (blue) or 4T1.13-miR-21-KD (red) mammary tumours (inoculum of 2 × 10^5^ cells) were culled on day 12 and the tumours collected for flow cytometry analysis of infiltrating immune cell lineages. For each immune cell profile, the mean ± SEM of 6 tumours is shown. The gating strategy is presented in Supplementary Fig. 3. **a** total CD45^+^ cells. **b** CD4^+^ T cells. **c** CD4^+^CD69^+^ T cells. **d** CD8^+^ T cells. **e** CD8^+^CD69^+^ T cells. **f** CD11b^+^Ly6G^+^ neutrophils. **g** CD11b^+^ly6c^lo^ monocytes. **h** CD11b^+^ly6c^lo^CD206^+^ monocytes. **i** CD11b^+^ly6c^lo^PD-L1^+^ monocytes. **j** CD11b^+^ly6c^hi^ monocytes. **k** CD11b^+^ly6c^hi^CD206^+^ monocytes. **l** CD11b^+^ly6c^hi^PD-L1^+^ monocytes.

To explore the mechanisms of potential immune suppression induced by miR-21, we assessed the extent of immune cell infiltration into 4T1.13-VC and 4T1.13-miR-21-KD tumours by flow cytometry and immunohistochemistry. Tumours were analysed 12 days after tumour cell inoculation, at a stage when regression was first evident in the miR-21-KD tumours (Fig. S2d, e). The flow cytometry gating strategy is shown in Fig. S3. Loss of miR-21 activity increased the overall number of CD45^+^ leucocytes (Fig. 4a), with corresponding increases in total and activated (CD69^+^) CD4^+^ and CD8^+^ T cells (Fig. 4b–e). In addition, the mean fluorescence intensity of CD69 was increased in the CD4^+^ and CD8^+^ T cells (Fig. S4a). The increases in total CD45^+^ cells, CD4^+^ and CD8^+^ T cells were confirmed by immunohistochemistry (Fig. S5).

Analysis of components of the innate immune system revealed no change in the overall number of neutrophils (Fig. 4f). However, reduced miR-21 activity led to diminished monocyte infiltration (Fig. 4g, j), particularly in Ly6C^hi^ inflammatory monocytes, with reduced numbers of Ly6C^hi^ but not Ly6C^lo^ monocytes expressing CD206, a marker of an M2-like polarisation state (Fig. 4h, k). In parallel, there were reduced numbers of both Ly6C^hi^ and Ly6C^lo^ monocytes expressing PD-L1 (Fig. 4i, l). There was no significant change in total natural killer (NK) cells, nor in their activation state as measured by CD69^+^ expression (Fig. S4a–c). The combination of an enhanced adaptive immune response and a reduction in PD-L1^+^ monocytes could explain the potent suppression of 4T1.13 tumour growth when miR-21 activity is reduced (Fig. 3c).

A similar analysis of the immune infiltrate of 66cl4 mammary tumours with or without enforced expression of miR-21 was completed. Tumours were recovered prior to miR-21-induced changes in growth rate (Fig. S6a, b). A trend towards decreased CD45^+^ cells was found (Fig. S6c), but no significant differences were found in the number of CD4^+^ and CD8^+^ T cells (Fig. S6d, e). However, elevated miR-21 did reduce the total number of NK and dendritic cells (DC) (Fig. S6f, g). Analysis of the myeloid compartment revealed no change in monocytes but a significant decrease in neutrophils (Fig. S6h–j). The reduction in type 1 effectors (NK, DC and CD8^+^ T cells), combined with the enhancement of metastasis of 66cl4-miR-21 tumours strongly indicates immune impairment as a contributing factor to increased metastasis and to increased tumour growth (Fig. 3a, b).

### Molecular mechanisms of miR-21 activity in tumour growth and metastasis

**Fig. 5:**
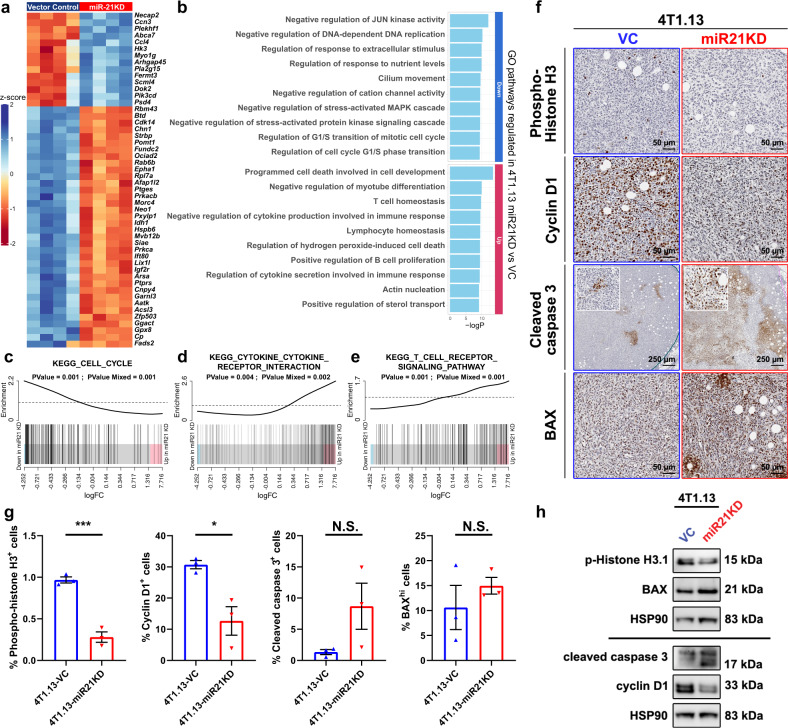
Gene expression profiling of whole primary tumours derived from 4T1.13-VC and 4T1.13-miR-21-KD cells. **a** Heat map showing the top 50 differentially expressed genes between 4T1.13-VC and 4T1.13-miR-21-KD tumours from RNA sequencing analysis. **b** Up-regulated and down-regulated Gene Ontology (GO) terms enriched in 4T1.13-miR-21-KD compared to 4T1.13-VC tumours, derived from the RNA sequencing data. **c**-**e** Barcode plots showing the significant down-regulation of genes involved in cell cycle activity (**c**), and upregulation of genes involved in cytokine-cytokine receptor interaction (**d**) and T cell receptor signalling (**e**) in the 4T1.13-miR-21-KD tumours. **f** IHC analysis of the levels of cell cycle- and apoptosis-related proteins in 4T1.13 tumours with or without miR-21 knockdown. Based on the bioinformatics analysis, cyclin D1 was down-regulated and BAX was up-regulated when miR-21 activity was reduced. **g** Quantitation of IHC staining levels in (**f**) using the Aperio digital pathology system. Mean ± SEM (*n* = 3 tumours/group). *p < 0.05; ***p < 0.001; N.S., not significant. **h** Western blot analysis of the expression of cell cycle- and apoptosis-related proteins in 4T1.13 cells cultured in vitro.

To further explore the molecular mechanisms through which miR-21 impacts tumour growth and metastasis, we analysed the transcriptome of primary tumours recovered 12 days after tumour cell inoculation when miR-21 knockdown tumours were beginning to regress (Fig. 3c). Four biological replicates of the 4T1.13-VC and miR-21-KD tumours were analysed by RNAseq, revealing ~4400 differentially expressed genes (FDR < 0.05) (Table S1), with the top 50 differentially expressed genes shown in Fig. 5a. Gene ontology (GO) analysis revealed down-regulation of terms relating to cell cycle and stress signalling and upregulation of cell death pathways and terms relating to increased immune activity when miR-21 levels were reduced (Fig. 5b). Gene set enrichment analysis confirmed significant down-regulation of genes involved in cell cycle activity (Fig. 5c), and upregulation of genes involved in cytokine-cytokine receptor interactions (Fig. 5d), T cell receptor signalling (Fig. 5e) and NK cell mediated cytotoxicity (Fig. S4d) in miR-21-KD tumours. These results are consistent with the observed decrease in 4T1.13-miR-21-KD tumour growth (Fig. 3c) and increased immune cell infiltration (Fig. 4).

To validate changes in cell cycle and cell death pathways, we immunostained primary 4T1.13 tumours with or without knockdown of miR-21 with relevant antibodies, demonstrating that loss of miR-21 reduced markers of cell cycle progression, namely phospho-histone H3 and cyclin D1 (Fig. 5f, g). Assessment of proteins involved in cell death, cleaved caspase 3 and BAX, showed a trend towards increased levels when miR-21 was reduced (Fig. 5f, g). When assessed in vitro, western blotting analysis of the levels of these proteins confirmed reduced cyclin D1 and phospho-histone H3 protein levels and increased caspase 3 processing, but no significant change in BAX levels was observed (Fig. 5h).

**Fig. 6:**
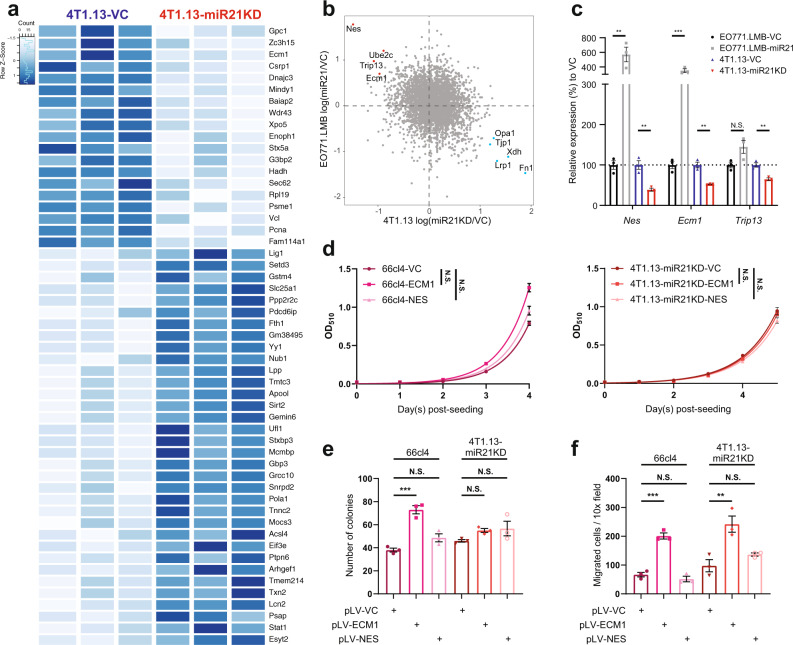
Proteomic analysis by mass spectrometry of EO771.lmb and 4T1.13 cells following modulation of miR-21 levels or activity. **a** Heatmap of the top differentially expressed proteins in 4T1.13-miR-21-KD cells compared to 4T1.13-VC cells. **b** Commonly up-regulated (red, upper left) and down-regulated (blue, lower right) proteins by miR-21 in the 4T1.13 and EO771.LMB cells. **c** qPCR validation of miR-21 targets identified in the mass spectrometry analysis. Mean ± SEM (*n* = 3). **d** Proliferation of 66cl4 and 4T1.13-miR-21KD cells with up-regulated expression of ECM1 or NES. Mean ± SEM (*n* = 6/group). **e** Colony formation of 66cl4 and 4T1.13-miR-21KD cells with up-regulated expression of ECM1 or NES. Mean ± SEM (*n* = 3/group). **f** Chemotactic migration of 66cl4 and 4T1.13-miR-21KD cells with up-regulated expression of ECM1 or NES. Mean ± SEM (*n* = 3/group). **p < 0.01; ***p < 0.001; N.S., not significant.

Since the second mechanism of action of miR-21 in the promotion of metastasis appeared to be independent of regulation by the immune system, we subjected both EO771.LMB and 4T1.13 cultured cells with and without modified miR-21 activity to proteomic analysis by mass spectrometry (Fig. 6a, Fig. S7). To analyse these data, we initially set out to complete pathway analysis using gene set enrichment analysis (GSEA). However, the level of expression of many proteins was low and this resulted in averaged protein changes that were often not significantly different between cells with high or low expression of miR-21. As an alternative analysis approach, we sought proteins that were consistently altered by miR-21 in the two different models. We did not expect a high correlation of protein expression changes between the two lines since 4T1.13 is a highly metastatic tumour in BALB/c mice and EO771.LMB is a mildly metastatic tumour in C57Bl/6 mice. In addition, the comparison was completed between miR-21 knockdown in 4T1.13 cells versus enforced miR-21 expression in EO771.LMB cells. However, this analysis revealed several proteins that responded similarly to changes in miR-21 in both cell lines, including nestin (NES), extracellular matrix protein-1 (ECM1) and thyroid receptor interacting protein 13 (TRIP13) that were all elevated in the presence of high miR-21 activity, while OPA1 levels were down-regulated in both lines (Fig. 6b). Assessment of transcript levels confirmed the changes in protein levels found for the three up-regulated genes, *Nes, Ecm1* and *Trip13* **(**Fig. 6c**)**.

To functionally validate these results, we generated both 66cl4 and 4T1.13-miR-21KD cells with exogenous expression of either NES or ECM1. Whilst neither gene conferred a growth advantage to the cells in vitro (Fig. 6d), ECM1 expression enhanced colony formation in the 66cl4 cells (Fig. 6e) and enhanced chemotactic migration of both cell lines (Fig. 6f).

## Discussion

In this study, modulation of miR-21 activity did not have a significant effect on proliferation in vitro, but consistent with other reports [24, 26, 48–50], we noted reduced colony formation and reduced chemotactic migration when miR-21 activity was low. Primary tumour growth in immunocompetent mice was severely impaired when miR-21 activity was suppressed. Analysis of the gene expression profiles of early-stage primary tumours confirmed the role of miR-21 in the promotion of cell division and replication, as predicted, but also indicated a role in the suppression of host anti-tumour immune responses. This observation was reinforced by the lack of impaired tumour growth in immunocompromised mice. Reduced miR-21 activity was associated with increased numbers and activation of CD4^+^ and CD8^+^ T cells, along with reduced numbers of infiltrating PD-L1^+^ monocytes. Consistent with our observations, in a cohort of colorectal cancer patients, tumour levels of miR-21 levels were inversely correlated with the density of CD3^+^ T cells, but surprisingly, not with CD8^+^ T cells [51]. In 66cl4 tumours, elevated miR-21 expression reduced the number of neutrophils as well as NK and DC cells. Thus, the means by which miR-21 alters the anti-tumour response may vary, depending on the individual tumour being assessed.

In line with our results, others have also shown that miR-21 is implicated in regulating the immune response to cancer, but have assessed the loss of stromal miR-21, not tumour-derived miR-21. Two reports using miR-21 null mice offer interesting but contradictory insights into immune alterations induced by miR-21. In preclinical models of hepatoma and fibrosarcoma, the absence of stromal miR-21 accelerated tumour growth compared to growth in wild type mice and was accompanied by reduced activation of CD4^+^ and CD8^+^ T cells [52]. MiR-21 appeared to enhance T cell activation after TCR/CD28 engagement by down-regulation of PTEN/AKT signalling, with reduced T cell activation when PTEN/AKT signalling was increased in the absence of host miR-21 [52]. It was shown previously that depletion of PTEN could enhance T cell activity after TCR/CD28 engagement [53]. However, in the second report, B16 and LLC1 tumours grew more slowly in miR-21 null mice despite the similar observation of reduced T cell activation, leading the authors to suggest that other factors were causing the anti-tumour response [54]. Enhanced polarisation of macrophages towards an anti-tumour M1-like phenotype was observed upon miR-21 depletion and co-injection of miR-21 null bone marrow derived monocytes after exposure to B16 cells in vitro resulted in reduced tumour growth [54]. Consistent with this, the reduced LLC tumour growth in miR-21 null mice could be replicated by selective depletion of miR-21 in macrophages, leading to enhanced cytotoxic T cell activity [55].

Cells of the immune system, in particular myeloid cells, have been shown to engulf exosomes, leading to the release of their contents and a resulting change in cell phenotype. For example, co-incubation of glioma-derived exosomes with stimulated mouse bone marrow cells led to induction of myeloid-derived suppressor cells (MDSC) with the ability to suppress CD8^+^ T cell proliferation. Exosomes from glioma cells depleted of miR-21 had reduced capacity to induce MDSC and suppress CD8^+^ T cell activation [56]. Uptake of glioma-derived exosomes by microglia has also been demonstrated in vivo, with microglia and macrophages from tumour-bearing brains revealed to have elevated levels of miR-21 compared to those from control brain tissue [57]. Thus, the miR-21 mediated suppression of the anti-tumour immune response that we have reported here could be mediated through release of miR-21 containing exosomes that are subsequently taken up by macrophages, thereby enhancing their pro-tumour activity.

Our study, focused on manipulation of tumour cell expression of miR-21, also revealed a second, apparently immune cell-independent role for miR-21 in promotion of metastasis. To investigate this further, we analysed the proteome of two tumour cell lines before and after enforced expression of miR-21. Three proteins, NES, ECM1 and TRIP13 were found in both lines to be up-regulated by miR-21, whilst OPA1 was down-regulated in both settings. The transcripts of these proteins do not appear to be direct targets of miR-21, as they do not contain the miR-21 consensus binding motif.

Nestin (NES) is an intermediate filament protein expressed by cancer cells, in particular by cancer stem cells [58] and is associated with poor prognosis in patients with triple negative breast cancer [59]. We could not detect any responses of cells in culture to changes in NES expression. ECM1 is an extracellular matrix protein that promotes angiogenesis and regulates the actin cytoskeleton [60]. ECM1 protein levels are higher in breast cancer samples than in normal breast epithelium and have been associated with increased lymphatic microvessel density in breast cancer patients [61]. Enforced ECM1 expression promoted colony formation and chemotactic migration of tumour lines. Thyroid hormone interacting protein 13 (TRIP13) regulates processes associated with mitosis and DNA repair. Expression of TRIP13 is elevated in tumours compared to the corresponding normal tissue and is positively correlated with poor outcome in multiple types of cancer [62]. We can therefore speculate that miR-21 might promote spontaneous metastasis through an immune cell independent mechanism possibly involving NES, ECM1 and/or TRIP13. The *OPA1* gene encodes the optic atrophy 1 protein, a dynamin GTPase involved in mitochondrial fusion and its knockdown in liver cancer inhibited tumour growth in mice [63]. Hence the down-regulation of OPA1 by miR-21 in our breast cancer lines is not consistent with this response in liver cancer.

Since miR-21 is up-regulated in a variety of cancer types and shown to drive tumour progression, there is much interest in it as a therapeutic target using anti-miRs, often in a nanoparticle format [64]. Given the observations of possible suppression of the anti-tumour immune response in mice null for miR-21 [52], targeted delivery to the tumour may be required, for example, by coupling a miR-21 antisense sequence to an EGFR aptamer, as described by Shu and colleagues [65].

In summary, we have demonstrated here that miR-21 in tumour cells acts through two separate mechanisms to drive tumour growth and metastasis. By the suppression of the anti-tumour immune response, miR-21 allows tumour growth and through a cell intrinsic mechanism, also promotes spontaneous metastasis. Thus effective targeting of tumour-derived miR-21 has potential to markedly suppress breast cancer progression, likely in combination with standard-of-care chemotherapy or radiotherapy.

## Materials and methods

### Cell culture

The 4T1 series of BALB/c murine mammary carcinoma cells, including 66cl4 and 4T1.13, have been described previously [45, 46]. AT-3 [66] and PyMT [67] are mammary tumour cell lines derived from the MMTV-PyMT transgenic mouse on a C57BL/6 background. H2N100 [68] and TA93 [69] are ERB-B2 over-expressing mammary tumour lines. B16.F10 melanoma, LLC lung carcinoma and RAW264.7 macrophages were obtained from ATCC. LM3, a mouse mammary tumour line was a kind gift from Alejandro Urtreger [70]. The EO771.LMB murine mammary carcinoma cell line was isolated from parental EO771 cells, as described previously [46]. Cells were cultured at 37 ^o^C in 5% CO_2_ (v/v) and were routinely tested for mycoplasma contamination. As these are all murine cell lines, standard STR was not able to be completed.

### MicroRNA array

miRNA profiling of 67NR and 4T1.2 cell lines was conducted using Exiqon miRCURY LNA microarrays (Qiagen, Australia). Briefly, total cell RNA (2 μg) isolated using Trizol (ThermoFisher) was labelled with 1 µL of Cy3 or Cy5 (500 ng/μL) fluorescent dinucleotide (Dharmacon, UK) in a buffer containing 0.1 mM ATP, 50 nM HEPES (pH 7.8), 2.5 mM DTT, 20 mM MgCl_2_, 10 mg/mL BSA, and 10% DMSO with 20 units RNA ligase (New England Biolabs) for 2 h at 4 °C. RNA was precipitated with 0.3 M sodium acetate (pH 5.2), 20 µg glycogen and 75% ethanol, washed with 70% ethanol, mixed and co-hybridised to the array in duplicate dye-swapping reactions at 60 °C overnight. Following washing, slides were analysed with a GenePix 4000B Scanner (Molecular Devices, USA) and differential expression analysed in Limma [71], using tools for linear modelling and the empirical Bayes statistic calculations.

### Generation of stably transduced cell lines

66cl4-mCherry and EO771.LMB-mCherry cells were transduced with pMSCVpuro-miR-21 (a gift from Andrei Goga (University of California, San Francisco) or empty vector retrovirus and selected for stable integration using puromycin (10μg/ml). Stably transduced cells were maintained in puromycin (5μg/ml). Knockdown of miR-21 function in 4T1.13 cells was achieved using the miArrest^TM^ lentiviral vector system (pEZX-AM03 vector backbone, GeneCopoeia, Rockville, MD, USA) that features the mCherry fluorescent reporter, the hygromycin B resistance gene, and a mature miR-21 sequestering RNA sequence driven by the H1 promoter (cat # MmiR-AN0316-AM03). The pEZX-AM03 vector expressing a scrambled non-sequestering RNA sequence was used as a negative control (cat # CmiR-AN0001-AM03). Pseudotyped lentiviral particles were transduced into 4T1.13 cells, subsequently selected with hygromycin B (800μg/ml, Sigma Aldrich), followed by single cell cloning by flow cytometry (FACSAriaII, BD Biosciences, Scoresby, Vic, Australia). Individual mCherry positive vector control (*n* = 6) or miR-21 knockdown (*n* = 6) clones were expanded and pooled for use. The resulting pooled cell populations were maintained in hygromycin B (400μg/ml). Stable expression of NES and ECM1 in the 66cl4 and 4T1.13-miR-21KD lines was achieved by lentiviral infection. Lentiviral constructs were generated by cloning the coding sequence of *Nes* or *Ecm1* (BC062893 or BC138693 from the Mammalian Gene Collection) into the pLV-EF1a-IRES-Puro backbone (Addgene 85132), with the addition of a Kozak sequence prior to the start codon.

### Proliferation and colony forming assay

Sulforhodamine B (SRB) proliferation assays were completed as described previously. For colony formation, cells (150/well) were seeded into 6-well or 12-well plates and incubated for 8 to14 days in full medium, followed by fixation and staining in 0.1% crystal violet in 50% methanol. The number of colonies (>50 cells) was scored for each well.

### Migration and invasion assays

Chemotactic migration and invasion assays were conducted in triplicate using Fluoroblok Transwells (BD Biosciences), as described previously [46]. Five fields per well and three triplicate wells at 10x magnification were scored.

### Quantitative RT-PCR (RT-qPCR)

Isolation of total RNA and analysis by RT-qPCR was conducted as described previously [46]. Residual DNA was removed using the TURBO DNA-free kit (Thermo Fischer). For detection of mature miR-21 levels, TaqMan stem-loop qPCR was used (Thermo Fisher Scientific, Scoresby, Vic, Australia) as described previously [72]. U6 snRNA was used as an internal reference gene (miRBase ID: 715680, Assay ID: 001973). For detection of protein-encoding mRNAs, single stranded cDNA was generated using ProtoScript II reverse transcriptase (New England Biolabs, Australia) and random pentadecamers. Details are presented in the Supplementary Methods.

### Western blotting

Details are presented in the Supplementary Methods.

### MiR-21 regulated reporter gene assay

To determine miR-21 functional activity in 66cl4 and EO771.LMB cell lines, a miR-21 reporter gene (kind gift from Andrei Goga, University of California San Francisco) containing the full-length human *PDCD4* 3′ UTR sequence cloned downstream of Firefly luciferase in the pGL3 control vector (Promega, Australia) was used in a dual luciferase assay in conjunction with the pRL-CMV internal control vector (Promega). Reporter gene activity was calculated by dividing Firefly luciferase activity by Renilla luciferase activity. To determine miR-21 functional activity in 4T1.13 cell lines, a miR-21 specific reporter was created by cloning a miR-21 binding site into XhoI/NotI restriction site downstream of the Renilla luciferase gene in the psiCHECK-2 vector (Promega). A separate Firefly luciferase cassette allowed for normalisation of the signal. psiCHECK-2 reporter gene activity was calculated by dividing Renilla luciferase activity by Firefly luciferase activity. Cell lines were transfected in 24-well plate format with plasmid DNA (1.0μg) using Lipofectamine 2000 (Thermo Fisher). Cells were lysed after 72 h and luciferase assays conducted using a Polarstar Optima with automatic substrate injection (BMG Labtech, Australia).

### Analysis of tumour growth and metastasis in vivo

All mouse experiments were approved by the Peter MacCallum Cancer Centre Animal Ethics Committee or the Austin Health Animal Ethics Committee prior to commencement. Female BALB/c, C57BL/6 and NOD.Cg-Prkdcscid Il2rgtm1Wjl/SzJ (NSG) mice were obtained from the Walter and Eliza Hall Institute, Australia. Female NOD.CB17-Prkdcscid/Arc (NOD.scid) and BALB/c-Foxn1nu/Arc (BALB/c nu/nu) mice were obtained from Animal Resources Centre, WA, Australia. Animals were housed in a clean and temperature-regulated facility with food and water *ad libitum*. Mouse numbers in each cohort were selected based on our experience and knowledge of the growth patterns of these tumours. Specific mouse numbers in each cohort are provided in figure legends. When mice were 7-8 weeks of age, the mice were randomised into two cohorts to receive orthotopic mammary tumours with or without modified expression of miR-21 in the inguinal mammary gland, with the number of cells injected and the strain of mice used detailed in the figure legends. Tumour volumes were measured using an electronic calliper, calculated as 0.5 × (length × width^2^). Although a rare event, for the analysis of tumour growth and metastasis, mice that did not develop tumours following tumour cell injection were excluded from each group. In some experiments, primary tumours were surgically resected at predetermined tumour volumes. Mice were closely monitored for signs of ill-health due to the development of metastatic disease and were humanely euthanized at ethical endpoint. The investigator was not blinded to the allocation of tumours to the mice. Lung metastatic burden was determined by multiplexed TaqMan genomic PCR as described previously [45] by calculating the ratio of mCherry gene amplification (derived from tumour cell genomic DNA) to mouse vimentin gene amplification (present in all cells).

### RNA-sequencing analysis

4T1.13-VC and 4T1.13-miR-21-KD orthotopic tumours (*n* = 4 per line) were recovered at day 12 as regression of 4T1.13-miR-21-KD tumours commenced. Total RNA was isolated and prepared for RNA sequencing. Details are provided in the Supplementary Methods.

### Proteomic analysis

#### In gel digestion

Equal amounts of whole-cell lysates were loaded onto precast NuPAGE^®^ 4–12% Bis-Tris gels in 1x MES SDS running buffer. Gels were run at a constant voltage of 150 V followed by visualisation of proteins with Coomassie stain (Bio-Rad). Gel bands [20] were excised and subjected to in-gel reduction, alkylation and trypsinization as described previously [73]. Details of the method are reported in the Supplementary Methods.

#### LC-MS/MS

Samples were analysed by LC-MS/MS using Q-Exactive plus and Fusion Lumos Orbitrap mass spectrometers (Thermo Scientific), both fitted with nanoflow reversed-phase-HPLC (Ultimate 3000 RSLC, Dionex). Details of the method are reported in the Supplementary Methods, with raw data presented in Table S3.

Database searching, protein identification and label-free spectral counting are described in Supplementary Methods.

### Immune profiling by flow cytometry

4T1.13-VC and 4T1.13-miR-21-KD tumours were recovered and weighed 12 days post-injection. 66cl4-VC and 66cl4-miR-21 tumours were recovered and weighed 16 days post-injection. Tumours were minced manually into fine pieces and dissociated in 1 mg/mL collagenase IV at 37 °C for 1 h. Red blood cells were lysed in 155 mM NH_4_Cl, 10 mM KHCO_3_ and 0.1 mM EDTA at room temperature for 5 min. Lysis reaction was stopped by adding 1 volume of PBS to the suspension. Cells were filtered and incubated with purified rat anti-mouse CD16/CD32 Fc block (BD Biosciences) at 4 °C for 30 min and stained with fluorochrome-conjugated antibodies recognising different immune cell markers (Table S2). For 4T1.13 tumours, panel one comprised CD45, TCRβ, CD8, CD4, CD69 and CD49b. Panel two comprised CD45, CD11b, Ly6c, Ly6g, CD206 and PD-L1 (CD274). For 66cl4 tumours, panel one comprised CD45, TCRβ, CD8, CD4, CD49b and NKp46. Panel two comprised CD45, CD11b, Ly6c, Ly6g, CD11c and MHCII). Cells were washed and analysed using a FACSAria II system.

### Immunohistochemistry

Primary tumours were fixed and immunostained for CD45, CD4, CD8, phospho-histone H3, cyclin D1, cleaved caspase 3 and BAX protein levels. Details of the method are reported in the Supplementary Methods.

### Breast cancer database analysis

Amplification of the miR-21 gene locus at 17q23 and its association with histological tumour grade were assessed from the METABRIC breast cancer patient dataset [74] in cBioPortal [75]. The association of miR-21 expression in primary breast tumours with overall patient survival was assessed from METABRIC (*n* = 1262) [74] and TCGA (*n* = 1078) [76] breast cancer patient datasets using the online Kaplan-Meier plotter (www.kmplot.com) with an automatically selected best cutoff [77].

### Data analysis

The means of two groups were compared using the Student’s *t* test, whereas comparison of more than two groups was completed using analysis of variance (ANOVA). Analyses were completed using GraphPad Prism v5. *p* < 0.05 was taken as statistically significant.

## Supplementary information

Supplemental Material

Supplementary Table S3

## Data Availability

The sequencing data were deposited into the Gene Expression Omnibus (GEO) with Accession No. GSE197520 (http://www.ncbi.nlm.nih.gov/geo/).

## References

[1] Ma L, Teruya-Feldstein J, Weinberg RA (2007). Tumour invasion and metastasis initiated by microRNA-10b in breast cancer. Nature.

[2] Kong W, Yang H, He L, Zhao JJ, Coppola D, Dalton WS (2008). MicroRNA-155 is regulated by the transforming growth factor beta/Smad pathway and contributes to epithelial cell plasticity by targeting RhoA. Mol Cell Biol.

[3] Huang Q, Gumireddy K, Schrier M, le Sage C, Nagel R, Nair S (2008). The microRNAs miR-373 and miR-520c promote tumour invasion and metastasis. Nat Cell Biol.

[4] Nurzadeh M, Naemi, M, Hasani, SS. A comprehensive review of oncogenic liRNAs in breast cancer. J Genet. 2021;100:15. 33764337

[5] Li X, Roslan S, Johnstone CN, Wright JA, Bracken CP, Anderson M (2014). MiR-200 can repress breast cancer metastasis through ZEB1-independent but moesin-dependent pathways. Oncogene..

[6] Dangi-Garimella S, Yun J, Eves EM, Newman M, Erkeland SJ, Hammond SM (2009). Raf kinase inhibitory protein suppresses a metastasis signalling cascade involving LIN28 and let-7. EMBO J.

[7] Tavazoie SF, Alarcon C, Oskarsson T, Padua D, Wang Q, Bos PD (2008). Endogenous human microRNAs that suppress breast cancer metastasis. Nature..

[8] Kong YW, Ferland-McCollough D, Jackson TJ, Bushell M (2012). microRNAs in cancer management. Lancet Oncol.

[9] Redvers RP, Anderson RL. Long non-coding RNA: agent provocateur in breast cancer metastasis. In: Jandial R, editor. Metastatic cancer: clinical and biological perspectives: Landes Bioscience; 2013. p. 178–97.

[10] Weidle UH, Birzele F, Kollmorgen G, Ruger R (2017). Long non-coding RNAs and their role in metastasis. Cancer Genomics Proteom.

[11] Cantile M, Di Bonito M, Cerrone M, Collina F, De Laurentiis M, Botti G. Long non-coding RNA HOTAIR in breast cancer therapy. Cancers (Basel). 2020;12:1197. 10.3390/cancers12051197 PMC7281113 32397382

[12] Pfeffer SR, Yang CH, Pfeffer LM (2015). The Role of miR-21 in Cancer. Drug Dev Res.

[13] Volinia S, Calin GA, Liu CG, Ambs S, Cimmino A, Petrocca F (2006). A microRNA expression signature of human solid tumors defines cancer gene targets. Proc Natl Acad Sci USA.

[14] Chen L, Li Y, Fu Y, Peng J, Mo MH, Stamatakos M (2013). Role of deregulated microRNAs in breast cancer progression using FFPE tissue. PLoS ONE.

[15] Lampis A, Hahne JC, Gasparini P, Cascione L, Hedayat S, Vlachogiannis G (2021). MIR21-induced loss of junctional adhesion molecule A promotes activation of oncogenic pathways, progression and metastasis in colorectal cancer. Cell Death Differ.

[16] Xu F, Xu L, Wang M, An G, Feng G (2015). The accuracy of circulating microRNA-21 in the diagnosis of colorectal cancer: a systematic review and meta-analysis. Colorectal Dis.

[17] Wang B, Zhang Q (2012). The expression and clinical significance of circulating microRNA-21 in serum of five solid tumors. J Cancer Res Clin Oncol.

[18] Anwar SL, Sari DNI, Kartika AI, Fitria MS, Tanjung DS, Rakhmina D (2019). Upregulation of circulating MiR-21 expression as a potential biomarker for therapeutic monitoring and clinical outcome in breast cancer. Asian Pac J Cancer Prev.

[19] Jinling W, Sijing S, Jie Z, Guinian W (2017). Prognostic value of circulating microRNA-21 for breast cancer: a systematic review and meta-analysis. Artif Cells Nanomed Biotechnol.

[20] Papadaki C, Stratigos M, Markakis G, Spiliotaki M, Mastrostamatis G, Nikolaou C (2018). Circulating microRNAs in the early prediction of disease recurrence in primary breast cancer. Breast Cancer Res.

[21] Pan F, Mao H, Deng L, Li G, Geng P (2014). Prognostic and clinicopathological significance of microRNA-21 overexpression in breast cancer: a meta-analysis. Int J Clin Exp Pathol.

[22] Yan LX, Huang XF, Shao Q, Huang MY, Deng L, Wu QL (2008). MicroRNA miR-21 overexpression in human breast cancer is associated with advanced clinical stage, lymph node metastasis and patient poor prognosis. RNA.

[23] MacKenzie TA, Schwartz GN, Calderone HM, Graveel CR, Winn ME, Hostetter G (2014). Stromal expression of miR-21 identifies high-risk group in triple-negative breast cancer. Am J Pathol.

[24] Si ML, Zhu S, Wu H, Lu Z, Wu F, Mo YY (2007). miR-21-mediated tumor growth. Oncogene.

[25] Meng F, Henson R, Wehbe-Janek H, Ghoshal K, Jacob ST, Patel T (2007). MicroRNA-21 regulates expression of the PTEN tumor suppressor gene in human hepatocellular cancer. Gastroenterology.

[26] Guan C, Zhang L, Wang S, Long L, Zhou H, Qian S (2019). Upregulation of MicroRNA-21 promotes tumorigenesis of prostate cancer cells by targeting KLF5. Cancer Biol Ther.

[27] Zhu S, Wu H, Wu F, Nie D, Sheng S, Mo YY (2008). MicroRNA-21 targets tumor suppressor genes in invasion and metastasis. Cell Res.

[28] Wang P, Zou F, Zhang X, Li H, Dulak A, Tomko RJ (2009). microRNA-21 negatively regulates Cdc25A and cell cycle progression in colon cancer cells. Cancer Res.

[29] Yan LX, Wu QN, Zhang Y, Li YY, Liao DZ, Hou JH (2011). Knockdown of miR-21 in human breast cancer cell lines inhibits proliferation, in vitro migration and in vivo tumor growth. Breast Cancer Res.

[30] Sahay D, Leblanc R, Grunewald TG, Ambatipudi S, Ribeiro J, Clezardin P (2015). The LPA1/ZEB1/miR-21-activation pathway regulates metastasis in basal breast cancer. Oncotarget..

[31] Hatley ME, Patrick DM, Garcia MR, Richardson JA, Bassel-Duby R, van Rooij E (2010). Modulation of K-Ras-dependent lung tumorigenesis by MicroRNA-21. Cancer Cell.

[32] Dan T, Shastri AA, Palagani A, Buraschi S, Neill T, Savage JE, et al. miR-21 plays a dual role in tumor formation and cytotoxic response in breast tumors. Cancers (Basel). 2021;13:888. 10.3390/cancers13040888 PMC7924198 33672628

[33] Ma X, Kumar M, Choudhury SN, Becker Buscaglia LE, Barker JR, Kanakamedala K (2011). Loss of the miR-21 allele elevates the expression of its target genes and reduces tumorigenesis. Proc Natl Acad Sci USA.

[34] Asangani IA, Rasheed SA, Nikolova DA, Leupold JH, Colburn NH, Post S (2008). MicroRNA-21 (miR-21) post-transcriptionally downregulates tumor suppressor Pdcd4 and stimulates invasion, intravasation and metastasis in colorectal cancer. Oncogene ..

[35] Chen B, Chen X, Wu X, Wang X, Wang Y, Lin TY (2015). Disruption of microRNA-21 by TALEN leads to diminished cell transformation and increased expression of cell-environment interaction genes. Cancer Lett.

[36] Gabriely G, Wurdinger T, Kesari S, Esau CC, Burchard J, Linsley PS (2008). MicroRNA 21 promotes glioma invasion by targeting matrix metalloproteinase regulators. Mol Cell Biol.

[37] Gong C, Nie Y, Qu S, Liao JY, Cui X, Yao H (2014). miR-21 induces myofibroblast differentiation and promotes the malignant progression of breast phyllodes tumors. Cancer Res.

[38] Han M, Wang F, Gu Y, Pei X, Guo G, Yu C (2016). MicroRNA-21 induces breast cancer cell invasion and migration by suppressing smad7 via EGF and TGF-beta pathways. Oncol Rep.

[39] Zhu S, Si ML, Wu H, Mo YY (2007). MicroRNA-21 targets the tumor suppressor gene tropomyosin 1 (TPM1). J Biol Chem.

[40] Choi DS, Kim DK, Kim YK, Gho YS (2013). Proteomics, transcriptomics and lipidomics of exosomes and ectosomes. Proteomics..

[41] Hannafon BN, Trigoso YD, Calloway CL, Zhao YD, Lum DH, Welm AL (2016). Plasma exosome microRNAs are indicative of breast cancer. Breast Cancer Res.

[42] Huang W, Kang XL, Cen S, Wang Y, Chen X (2015). High-level expression of microRNA-21 in peripheral blood mononuclear cells is a diagnostic and prognostic marker in prostate cancer. Genet Test Mol Biomark.

[43] Peinado H, Zhang H, Matei IR, Costa-Silva B, Hoshino A, Rodrigues G (2017). Pre-metastatic niches: organ-specific homes for metastases. Nat Rev Cancer.

[44] Yuan X, Qian N, Ling S, Li Y, Sun W, Li J (2021). Breast cancer exosomes contribute to pre-metastatic niche formation and promote bone metastasis of tumor cells. Theranostics.

[45] Eckhardt BL, Parker BS, van Laar RK, Restall CM, Natoli AL, Tavaria MD (2005). Genomic analysis of a spontaneous model of breast cancer metastasis to bone reveals a role for the extracellular matrix. Mol Cancer Res.

[46] Johnstone CN, Smith YE, Cao Y, Burrows AD, Cross RS, Ling X (2015). Functional and molecular characterisation of EO771.LMB tumours, a new C57BL/6-mouse-derived model of spontaneously metastatic mammary cancer. Dis Model Mech.

[47] Lu Z, Liu M, Stribinskis V, Klinge CM, Ramos KS, Colburn NH (2008). MicroRNA-21 promotes cell transformation by targeting the programmed cell death 4 gene. Oncogene..

[48] Chu NJ, Anders RA, Fertig EJ, Cao M, Hopkins AC, Keenan BP (2020). Inhibition of miR-21 Regulates Mutant KRAS Effector Pathways and Intercepts Pancreatic Ductal Adenocarcinoma Development. Cancer Prev Res (Philos).

[49] Lei M, Xie W, Sun E, Sun Y, Tian D, Liu C (2015). microRNA-21 Regulates Cell Proliferation and Migration and Cross Talk with PTEN and p53 in Bladder Cancer. DNA Cell Biol.

[50] Wang H, Tan Z, Hu H, Liu H, Wu T, Zheng C (2019). microRNA-21 promotes breast cancer proliferation and metastasis by targeting LZTFL1. BMC Cancer.

[51] Mima K, Nishihara R, Nowak JA, Kim SA, Song M, Inamura K (2016). MicroRNA MIR21 and T cells in colorectal cancer. Cancer Immunol Res.

[52] He W, Wang C, Mu R, Liang P, Huang Z, Zhang J (2017). MiR-21 is required for anti-tumor immune response in mice: an implication for its bi-directional roles. Oncogene..

[53] Locke FL, Zha YY, Zheng Y, Driessens G, Gajewski TF (2013). Conditional deletion of PTEN in peripheral T cells augments TCR-mediated activation but does not abrogate CD28 dependency or prevent anergy induction. J Immunol.

[54] Xi J, Huang Q, Wang L, Ma X, Deng Q, Kumar M (2018). miR-21 depletion in macrophages promotes tumoricidal polarization and enhances PD-1 immunotherapy. Oncogene..

[55] Sahraei M, Chaube B, Liu Y, Sun J, Kaplan A, Price NL (2019). Suppressing miR-21 activity in tumor-associated macrophages promotes an antitumor immune response. J Clin Invest.

[56] Guo X, Qiu W, Liu Q, Qian M, Wang S, Zhang Z (2018). Immunosuppressive effects of hypoxia-induced glioma exosomes through myeloid-derived suppressor cells via the miR-10a/Rora and miR-21/Pten Pathways. Oncogene..

[57] van der Vos KE, Abels ER, Zhang X, Lai C, Carrizosa E, Oakley D (2016). Directly visualized glioblastoma-derived extracellular vesicles transfer RNA to microglia/macrophages in the brain. Neuro Oncol.

[58] Tampaki EC, Nakopoulou L, Tampakis A, Kontzoglou K, Weber WP, Kouraklis G (2014). Nestin involvement in tissue injury and cancer—a potential tumor marker?. Cell Oncol (Dordr).

[59] Piras F, Ionta MT, Lai S, Perra MT, Atzori F, Minerba L (2011). Nestin expression associates with poor prognosis and triple negative phenotype in locally advanced (T4) breast cancer. Eur J Histochem.

[60] Gomez-Contreras P, Ramiro-Diaz JM, Sierra A, Stipp C, Domann FE, Weigel RJ (2017). Extracellular matrix 1 (ECM1) regulates the actin cytoskeletal architecture of aggressive breast cancer cells in part via S100A4 and Rho-family GTPases. Clin Exp Metastasis.

[61] Wu QW, She HQ, Liang J, Huang YF, Yang QM, Yang QL (2012). Expression and clinical significance of extracellular matrix protein 1 and vascular endothelial growth factor-C in lymphatic metastasis of human breast cancer. BMC Cancer.

[62] Lu S, Qian J, Guo M, Gu C, Yang Y (2019). Insights into a Crucial Role of TRIP13 in Human Cancer. Comput Struct Biotechnol J..

[63] Li M, Wang L, Wang Y, Zhang S, Zhou G, Lieshout R, et al. Mitochondrial fusion via OPA1 and MFN1 supports liver tumor cell metabolism and growth. Cells. 2020;9. 10.3390/cells9010121 PMC7017104 31947947

[64] Seo YE, Suh HW, Bahal R, Josowitz A, Zhang J, Song E (2019). Nanoparticle-mediated intratumoral inhibition of miR-21 for improved survival in glioblastoma. Biomaterials..

[65] Shu D, Li H, Shu Y, Xiong G, Carson WE, Haque F (2015). Systemic delivery of anti-miRNA for suppression of triple negative breast cancer utilizing RNA nanotechnology. ACS Nano.

[66] Stewart TJ, Abrams SI (2007). Altered immune function during long-term host-tumor interactions can be modulated to retard autochthonous neoplastic growth. J Immunol.

[67] Johnstone CN, Tu Y, Langenbach S, Baloyan D, Pattison AD, Lock P, et al. Annexin A1 is required for efficient tumor initiation and cancer stem cell maintenance in a model of human breast cancer. Cancers (Basel). 2021;13:1154. 10.3390/cancers13051154 PMC7962654 33800279

[68] Ma Y, Yamazaki T, Yang H, Kepp O, Galluzzi L, Zitvogel L (2013). Tumor necrosis factor is dispensable for the success of immunogenic anticancer chemotherapy. Oncoimmunology..

[69] Miao RY, Drabsch Y, Cross RS, Cheasley D, Carpinteri S, Pereira L (2011). MYB is essential for mammary tumorigenesis. Cancer Res.

[70] Urtreger A, Ladeda V, Puricelli L, Rivelli A, Vidal M, Delustig E (1997). Modulation of fibronectin expression and proteolytic activity associated with the invasive and metastatic phenotype in two new murine mammary tumor cell lines. Int J Oncol.

[71] Ritchie ME, Phipson B, Wu D, Hu Y, Law CW, Shi W (2015). limma powers differential expression analyses for RNA-sequencing and microarray studies. Nucleic Acids Res.

[72] Zhang L, Volinia S, Bonome T, Calin GA, Greshock J, Yang N (2008). Genomic and epigenetic alterations deregulate microRNA expression in human epithelial ovarian cancer. Proc Natl Acad Sci USA.

[73] Mathivanan S, Ji H, Tauro BJ, Chen YS, Simpson RJ (2012). Identifying mutated proteins secreted by colon cancer cell lines using mass spectrometry. J Proteomics.

[74] Curtis C, Shah SP, Chin SF, Turashvili G, Rueda OM, Dunning MJ (2012). The genomic and transcriptomic architecture of 2,000 breast tumours reveals novel subgroups. Nature.

[75] Cerami E, Gao J, Dogrusoz U, Gross BE, Sumer SO, Aksoy BA (2012). The cBio cancer genomics portal: an open platform for exploring multidimensional cancer genomics data. Cancer Disco.

[76] Cancer Genome Atlas N. (2012). Comprehensive molecular portraits of human breast tumours. Nature.

[77] Gyorffy B, Lanczky A, Eklund AC, Denkert C, Budczies J, Li Q (2010). An online survival analysis tool to rapidly assess the effect of 22,277 genes on breast cancer prognosis using microarray data of 1,809 patients. Breast Cancer Res. Treat.

