# Supplementary material for: MicroRNA-21 is immunosuppressive and pro-metastatic via separate mechanisms
Source: Oncogenesis. 2022 Jul 11;11(1):38. doi: 10.1038/s41389-022-00413-7 (PMC9276829; doi:10.1038/s41389-022-00413-7)
Supplement: Supplementary file 1 — Supplemental Material [file 41389_2022_413_MOESM1_ESM.pdf]

Supplementary Methods

Quantitative RT-PCR

Isolation of total RNA and analysis by RT-qPCR was conducted as described previously (1) Residual DNA was removed using the TURBO DNA-free kit (Thermo Fischer). For detection of mature miR-21 levels, TaqMan stem-loop qPCR was used (Thermo Fisher Scientific, Scoresby, Vic, Australia) as described previously (2). U6 snRNA was used as an internal reference gene (miRBase ID: 715680, Assay ID: 001973). For detection of protein-encoding mRNAs, single stranded cDNA was generated using ProtoScript II reverse transcriptase (New England Biolabs, Australia) and random pentadecamers.

Expression of mouse *Ecm1*, *Nes* and *Trip13* was determined using the SYBR Green real-time PCR master mix (Thermo Fischer) with ribosomal protein S27a (*Rps27a*) as an internal control. The oligonucleotide primer sequences used are as follows. *Ecm1*_F, 5' AAC CTG CCG CAG ACT GGC TA 3'; *Ecm1*_R, 5' GCA TCC TCC CAC ACA AGC TTC A 3'. *Nes*_F, 5' GCA GCA ACT GGC ACA CCT CAA 3'; *Nes*_R, 5' GCT TCA GCT TGG GGT CAG GAA A 3'. *Trip13*_F, 5' CCT GGA ACC GGG TGG TGC TG 3'; *Trip13*_R, 5' ATA CCG GTA CCT GCT CGA CAG TCT 3'. *Rps27a*_F, 5' CGG GGA AAA CCA TCA CGC TCG AGG TT 3'; *Rps27a*_R, CGG CCA TCT TCC AGC TGC TTA CCA GC 3'. PCR reactions were run for 45-50 cycles in 96-well Fast optical reaction plates using a StepOne-Plus or ViiA 7 real-time PCR system (Thermo Fisher).

Western blotting

Cells were lysed with cold lysis buffer (2% SDS and 50 mM Tris-HCl) and heated at 95 °C for 10 minutes. Large protein aggregates or genomic DNA were sheared by passing the lysates through a needle and syringe several times. Proteins were dissolved in 0.1 M DTT, 4% glycerol and 0.0004% bromophenol blue in lysis buffer) and subjected to gel electrophoresis and transferred to PVDF membranes (Millipore) using the Bolt system (Thermo Fisher). Membranes were blocked with 5% milk in PBS and incubated with primary antibodies (**Table S2**) at 4°C overnight. Following incubation with HRP-conjugated secondary antibodies, protein signals were detected using the enhanced chemiluminescence substrate (PerkinElmer). For assessment of multiple proteins on one membrane, immunostaining was done sequentially, knowing that each protein was located at a different molecular weight on the membrane. Antibodies used for each blot had been tested previously to demonstrate that each protein ran at a unique molecular weight. Images of the same blot were overlayed to ensure that bands produced by different antibodies did not overlap. When stripping was required to

reduce the intensity of previously stained bands, membranes were washed with warm stripping buffer (1.5% glycine, 0.1% SDS and 1% Tween 20, pH 2.2) four times for 5 minutes each time, followed by blocking and antibody detection.

RNA-Sequencing analysis

4T1.13-VC and 4T1.13-miR21KD orthotopic tumours (n = 4 per line) were recovered at day 14 as regression of 4T1.13-miR21KD tumours commenced. Total RNA was isolated using the Ambion miRVana total RNA isolation kit (Thermo Fisher). RNA was quantified using the Qubit RNA HS Assay Kit (Thermo Fisher) and 5μg treated with DNase I (Ambion Turbo DNA-free kit, Thermo Fisher). RNA-Seq was completed by the Australian Genome Research Facility (AGRF, Melbourne, Australia) on the Illumina HiSeq with 100bp SE sequencing using v4 chemistry, with 12 samples multiplexed in a single lane. Single-end 100bp reads were mapped to RefSeq mm10 genome build using subread (3). Read counts at the gene level were quantified using featureCounts (4). Gene counts with counts-per-million (cpm) larger than 0.5 in at least four samples were retained and normalized by TMM normalization (Robinson and Oshlack, 2010). Differential gene expression analysis was completed using the edgeR Quasi-likelihood Negative Binomial Generalized log-linear model framework (5). Genes with FDR<0.05 were determined as differentially expressed. Biological process-related gene ontology terms (annotated in the GO.db package) that are enriched in 4T1.13-miR21-KD tumours were identified using the *fry* function in the limma package.

Proteomic analysis

In gel digestion

Equal amounts of whole cell lysates were loaded onto precast NuPAGE® 4–12% Bis-Tris gels in 1x MES SDS running buffer. Gels were run at a constant voltage of 150 V followed by visualization of proteins with Coomassie stain (Bio-Rad). Gel bands (20) were excised and subjected to in-gel reduction, alkylation and trypsinization as described previously (6). Briefly, gel bands were reduced with 10 mM DTT (Bio-Rad), alkylated with 25 mM iodoacetamide (Sigma) and digested overnight at 37ºC with 150 ng of sequencing grade trypsin (Promega). The tryptic peptides were extracted by 0.1% trifluoroacetic acid in 50% (w/v) acetonitrile.

LC-MS/MS

Samples were analysed by LC-MS/MS using Q-Exactive plus and Fusion Lumos Orbitrap mass spectrometers (Thermo Scientific), both fitted with nanoflow reversed-phase-HPLC (Ultimate 3000 RSLC, Dionex). The nano-HPLC system was equipped with an Acclaim Pepmap nano-

trap column (Dionex – C18, 100 Å, 75 µm × 2 cm) and an Acclaim Pepmap RSLC analytical column (Dionex – C18, 100 Å, 75 µm × 50 cm). Typically, for each LC-MS/MS experiment, 1µL of the peptide mix was loaded onto the enrichment (trap) column at an isocratic flow of 5µL/min of 3% CH3CN containing 0.1% formic acid for 5 min before the enrichment column was switched in-line with the analytical column. The eluents used for the LC were 0.1% (v/v) formic acid (solvent A) and 100% CH3CN/0.1% (v/v) formic acid (solvent B). The gradient used was 3% B to 25% B for 23 min, 25% B to 40% B in 2 min, 40% B to 85% B in 2 min and maintained at 85% B for 2 min before equilibration for 10 min at 3% B prior to the next injection. All spectra were acquired in positive mode with full scan MS spectra scanning from m/z 300–1650 in the FT mode at 70 000 (QE) and 120,000 (Lumos) resolution. Lockmass of 445.12003 m/z was used for both instruments. For MS/MS on the Lumos, the "top speed" acquisition method mode (3 sec cycle time) on the most intense precursor was used whereby peptide ions with charge states ≥2 were isolated with isolation window of 1.6 m/z and fragmented with HCD using normalized collision energy of 35. For MS/MS on the QE plus, the 15 most intense peptide ions with charge states ≥2 were isolated with isolation window of 1.6 m/z and fragmented by HCD with normalized collision energy of 35. Dynamic exclusion of 30s was applied.

Database searching, protein identification and label-free spectral counting

Peak lists were extracted from raw mass spectrometry into the Mascot Generic File Format (MGF) using MsConvert with peak picking. The MGF files were then searched using X! Tandem (Sledgehammer edition version 2013.09.01.1) against a NR protein database. Search parameters used were: fixed modification (carboamidomethylation of cysteine; +57 Da), variable modifications (oxidation of methionine; +16Da and N-terminal acetylation; +42 Da), two missed tryptic cleavages, 20 ppm peptide mass tolerance and 0.6Da fragment ion mass tolerance. Proteins were quantified using the Normalized Spectral Abundance Factor (NSAF) method (7, 8).

Immunohistochemistry

Fresh tumours were fixed in 4% paraformaldehyde at 4°C overnight, dehydrated and embedded in paraffin. Sections (4µm) were rehydrated and equilibrated in PBS-Tween 20 (PBS-T). Endogenous peroxidases were inactivated in 3% hydrogen peroxide for 10 min. Antigen retrieval was not required for anti-CD45, CD4 or CD8 antibodies. For rabbit cyclin D1, phospho-histone H3, cleaved caspase 3 and BAX antibodies, antigen retrieval was performed with citrate buffer (pH6) at sub-boiling temperature in a microwave for 12 minutes.

Information of the antibodies and their dilutions can be found in Supplementary Table 2. Sections were blocked with 3% normal goat serum in PBS-T at room temperature for 1h, followed by incubation with the primary antibodies in blocking buffer at 4°C overnight. Following incubation with biotin-conjugated goat anti-rat secondary antibodies (1:200 dilution) and the avidin-biotin complex (ABC) reagent (Vector Laboratories, Burlingame, CA, USA), sections were stained with 3,3' diaminobenzidine (DAB), counterstained with haematoxylin, and mounted in entellan.

Supplementary Data References

1. Johnstone CN, Smith YE, Cao Y, Burrows AD, Cross RS, Ling X, et al. Functional and molecular characterisation of EO771.LMB tumours, a new C57BL/6-mouse-derived model of spontaneously metastatic mammary cancer. Dis Model Mech. 2015;8(3):237-51.
2. Kong W, Yang H, He L, Zhao JJ, Coppola D, Dalton WS, et al. MicroRNA-155 is regulated by the transforming growth factor beta/Smad pathway and contributes to epithelial cell plasticity by targeting RhoA. Molecular and cellular biology. 2008;28(22):6773-84.
3. Liao Y, Smyth GK, Shi W. The Subread aligner: fast, accurate and scalable read mapping by seed-and-vote. Nucleic Acids Res. 2013;41(10):e108.
4. Liao Y, Smyth GK, Shi W. featureCounts: an efficient general purpose program for assigning sequence reads to genomic features. Bioinformatics. 2014;30(7):923-30.
5. Robinson MD, Oshlack A. A scaling normalization method for differential expression analysis of RNA-seq data. Genome Biol. 2010;11(3):R25.
6. Mathivanan S, Ji H, Tauro BJ, Chen YS, Simpson RJ. Identifying mutated proteins secreted by colon cancer cell lines using mass spectrometry. J Proteomics. 2012;76 Spec No.:141-9.
7. Samuel M, Fonseka P, Sanwlani R, Gangoda L, Chee SH, Keerthikumar S, et al. Oral administration of bovine milk-derived extracellular vesicles induces senescence in the primary tumor but accelerates cancer metastasis. Nat Commun. 2021;12(1):3950.
8. Paoletti AC, Parmely TJ, Tomomori-Sato C, Sato S, Zhu D, Conaway RC, et al. Quantitative proteomic analysis of distinct mammalian Mediator complexes using normalized spectral abundance factors. Proc Natl Acad Sci U S A. 2006;103(50):18928-33.

Supplementary Table Legends

Supplementary Table 1

Gene Ontology (GO) analysis of the transcriptomes of primary tumours derived from 4T1.13-VC and 4T1.13-miR-21-KD tumours.

Supplementary Table 2

Source of antibodies used in this study.

Supplementary Table 3

Proteins detected by mass spectrometry in three biological replicates of 4T1.13-miR-21-KD (4TM), 4T1.12-VC (4TV), EO771.LMB-miR-21 (EOM) and EO771.LMB-VC (EOV).

Supplementary Table Legends

Supplementary Table 1

Gene Ontology (GO) analysis of the transcriptomes of primary tumours derived from 4T1.13-VC and 4T1.13-miR-21-KD tumours.

Supplementary Table 2

Source of antibodies used in this study.

Supplementary Table 3

Proteins detected by mass spectrometry in three biological replicates of 4T1.13-miR-21-KD (4TM), 4T1.12-VC (4TV), EO771.LMB-miR-21 (EOM) and EO771.LMB-VC (EOV).

Supplementary Table 1

	NGenes	Direction	PValue	FDR	PValue.Mix	FDR.Mixed
GO_PROGRAMMED_CELL_DEATH_INVOLVED_IN_CELL_DEVELOPMENT	15	Up	1.31E-06	0.006761	0.000464	0.000502
GO_NEGATIVE_REGULATION_OF_JUN_KINASE_ACTIVITY	11	Down	5.7E-06	0.006761	2.26E-05	0.000302
GO_NEGATIVE_REGULATION_OF_DNA-DEPENDENT_DNA_REPLICATION	16	Down	3.62E-05	0.006761	5.51E-05	0.000302
GO_NEGATIVE_REGULATION_OF_MYOTUBE_DIFFERENTIATION	13	Up	4.74E-05	0.006761	1.86E-05	0.000302
GO_REGULATION_OF_RESPONSE_TO_EXTRACELLULAR_STIMULUS	17	Down	4.79E-05	0.006761	0.000179	0.000302
GO_REGULATION_OF_RESPONSE_TO_NUTRIENT_LEVELS	17	Down	4.79E-05	0.006761	0.000179	0.000302
GO_T_CELL_HOMEOSTASIS	41	Up	5.66E-05	0.006761	4.86E-05	0.000302
GO_CILIUM_MOVEMENT	33	Down	6.58E-05	0.006761	6.72E-05	0.000302
GO_NEGATIVE_REGULATION_OF_CYTOKINE_PRODUCTION_INVOLVED_IN_IMMUNE_RESPONSE	20	Up	7.11E-05	0.006761	8.6E-05	0.000302
GO_LYMPHOCYTE_HOMEOSTASIS	70	Up	7.28E-05	0.006761	7.09E-05	0.000302
GO_NEGATIVE_REGULATION_OF_CATION_CHANNEL_ACTIVITY	23	Down	7.36E-05	0.006761	4.84E-05	0.000302
GO_NEGATIVE_REGULATION_OF_STRESS-ACTIVATED_MAPK_CASCADE	37	Down	7.53E-05	0.006761	0.000125	0.000302
GO_NEGATIVE_REGULATION_OF_STRESS-ACTIVATED_PROTEIN_KINASE_SIGNALING_CASCADE	37	Down	7.53E-05	0.006761	0.000125	0.000302
GO_REGULATION_OF_HYDROGEN_PEROXIDE-INDUCED_CELL_DEATH	29	Up	7.81E-05	0.006761	0.000116	0.000302
GO_POSITIVE_REGULATION_OF_B_CELL_PROLIFERATION	39	Up	7.9E-05	0.006761	0.000125	0.000302
GO_REGULATION_OF_G1/S_TRANSITION_OF_MITOTIC_CELL_CYCLE	102	Down	7.92E-05	0.006761	0.000203	0.000308
GO_REGULATION_OF_CELL_CYCLE_G1/S_PHASE_TRANSITION	115	Down	8.42E-05	0.006761	0.000203	0.000308
GO_REGULATION_OF_CYTOKINE_SECRETION_INVOLVED_IN_IMMUNE_RESPONSE	13	Up	8.55E-05	0.006761	0.0002	0.000307
GO_CILIUM_OR_FLAGELLUM-DEPENDENT_CELL_MOTILITY	16	Down	9.54E-05	0.006761	0.000104	0.000302
GO_CILIUM-DEPENDENT_CELL_MOTILITY	16	Down	9.54E-05	0.006761	0.000104	0.000302
GO_ACTIN_NUCLEATION	42	Up	0.000107	0.006761	9.79E-05	0.000302
GO_POSITIVE_REGULATION_OF_STEROL_TRANSPORT	19	Up	0.000108	0.006761	0.000103	0.000302
GO_POSITIVE_REGULATION_OF_CHOLESTEROL_TRANSPORT	19	Up	0.000108	0.006761	0.000103	0.000302
GO_CELL-CELL_ADHESION_MEDIATED_BY_INTEGRIN	11	Up	0.000108	0.006761	2.92E-05	0.000302
GO_POSITIVE_REGULATION_OF_INTERLEUKIN-8_SECRETION	12	Up	0.000108	0.006761	0.000122	0.000302
GO_POSITIVE_REGULATION_OF_CHOLESTEROL_EFFLUX	12	Up	0.000112	0.006761	5.89E-05	0.000302
GO_POSITIVE_REGULATION_OF_HISTONE_DEACETYLATION	19	Up	0.000112	0.006761	2.56E-05	0.000302
GO_NEGATIVE_REGULATION_OF_CALCIUM_ION_TRANSPORT	37	Down	0.000121	0.006761	6.85E-05	0.000302
GO_NEGATIVE_REGULATION_OF_PHOSPHATIDYLINOSITOL_3-KINASE_SIGNALING	13	Down	0.000127	0.006761	8.52E-05	0.000302
GO_REGULATION_OF_CHOLESTEROL_EFFLUX	15	Up	0.00013	0.006761	7.5E-05	0.000302
GO_WATER-SOLUBLE_VITAMIN_METABOLIC_PROCESS	26	Down	0.00013	0.006761	5.35E-05	0.000302
GO_MAINTENANCE_OF_PROTEIN_LOCATION_IN_NUCLEUS	22	Down	0.000134	0.006761	2.88E-05	0.000302
GO_MACROPHAGE_CHEMOTAXIS	34	Up	0.000135	0.006761	5.67E-05	0.000302
GO_DEVELOPMENT_OF_SECONDARY_SEXUAL_CHARACTERISTICS	11	Up	0.000135	0.006761	3.68E-05	0.000302
GO_PLATELET_FORMATION	16	Up	0.000139	0.006761	0.00028	0.000351
GO_CELLULAR_RESPONSE_TO_GRANULOCYTE_MACROPHAGE_COLONY-STIMULATING_FACTOR_STIMULUS	11	Up	0.000146	0.006761	0.000168	0.000302
GO_HYPERSENSITIVITY	11	Up	0.000147	0.006761	0.000151	0.000302
GO_POSITIVE_REGULATION_OF_SMOOTHENED_SIGNALING_PATHWAY	30	Down	0.000149	0.006761	0.000228	0.000322
GO_POSITIVE_REGULATION_OF_LEUKOCYTE_APOPTOTIC_PROCESS	34	Up	0.000153	0.006761	6.17E-05	0.000302
GO_RESPONSE_TO_FUNGUS	31	Up	0.000155	0.006761	0.000128	0.000302
GO_EOSINOPHIL_CHEMOTAXIS	19	Up	0.000157	0.006761	0.000106	0.000302
GO_POSITIVE_REGULATION_OF_LYMPHOCYTE_APOPTOTIC_PROCESS	22	Up	0.000159	0.006761	4.51E-05	0.000302
GO_CILIUM_ORGANIZATION	243	Down	0.000161	0.006761	0.000127	0.000302
GO_NEGATIVE_REGULATION_OF_MYELOID_CELL_APOPTOTIC_PROCESS	16	Up	0.000163	0.006761	0.000105	0.000302
GO_MACROPHAGE_MIGRATION	38	Up	0.000164	0.006761	6.59E-05	0.000302
GO_CELLULAR_RESPONSE_TO_INTERLEUKIN-1	65	Up	0.000164	0.006761	0.000103	0.000302
GO_POSITIVE_REGULATION_OF_B_CELL_DIFFERENTIATION	16	Up	0.000164	0.006761	0.000102	0.000302
GO_RESPONSE_TO_INTERLEUKIN-1	78	Up	0.000165	0.006761	8.04E-05	0.000302
GO_CILIUM_ASSEMBLY	227	Down	0.000165	0.006761	0.000122	0.000302
GO_RESPONSE_TO_LIGHT_STIMULUS	215	Down	0.00017	0.006761	0.000148	0.000302
GO_RETROGRADE_TRANSPORT,_ENDOSOME_TO_PLASMA_MEMBRANE	19	Down	0.00017	0.006761	4.37E-05	0.000302
GO_RESPONSE_TO_GRANULOCYTE_MACROPHAGE_COLONY-STIMULATING_FACTOR	12	Up	0.000173	0.006761	0.000136	0.000302
GO_RESPONSE_TO_MURAMYL_DIPEPTIDE	15	Up	0.000173	0.006761	1.12E-05	0.000302
GO_REGULATION_OF_RESPONSE_TO_REACTIVE_OXYGEN_SPECIES	38	Up	0.000182	0.006761	0.000157	0.000302
GO_B_CELL_PROLIFERATION	73	Up	0.000185	0.006761	0.000189	0.000305
GO_REGULATION_OF_T_CELL_CYTOKINE_PRODUCTION	22	Up	0.000189	0.006761	0.000113	0.000302
GO_ANTIBIOTIC_BIOSYNTHETIC_PROCESS	12	Up	0.000189	0.006761	9.1E-05	0.000302
GO_FC_RECEPTOR_MEDIATED_STIMULATORY_SIGNALING_PATHWAY	11	Up	0.00019	0.006761	0.000112	0.000302
GO_REGULATION_OF_MYOTUBE_DIFFERENTIATION	43	Up	0.000191	0.006761	8.07E-05	0.000302
GO_SPINAL_CORD_PATTERNING	15	Down	0.000195	0.006761	0.000227	0.000321
GO_OLIGOSACCHARIDE-LIPID_INTERMEDIATE_BIOSYNTHETIC_PROCESS	13	Down	0.000196	0.006761	6.97E-05	0.000302
GO_NEGATIVE_REGULATION_OF_PROTEIN_SECRETION	102	Up	0.000197	0.006761	8.22E-05	0.000302
GO_MITOCHONDRIAL_CALCIUM_ION_HOMEOSTASIS	24	Up	0.000198	0.006761	8.56E-06	0.000302
GO_EOSINOPHIL_MIGRATION	22	Up	0.000202	0.006761	0.000124	0.000302
GO_DEFENSE_RESPONSE_TO_FUNGUS	20	Up	0.000203	0.006761	9.96E-05	0.000302
GO_B_CELL_HOMEOSTASIS	36	Up	0.000205	0.006761	0.000139	0.000302
GO_REGULATION_OF_CYTOKINE_PRODUCTION_INVOLVED_IN_IMMUNE_RESPONSE	60	Up	0.000207	0.006761	0.00014	0.000302
GO_PROTEIN_MANNOSYLATION	15	Down	0.000208	0.006761	0.000162	0.000302
GO_CELL_DEATH_IN_RESPONSE_TO_HYDROGEN_PEROXIDE	31	Up	0.000209	0.006761	0.000129	0.000302
GO_MOTILE_CILIUM_ASSEMBLY	15	Down	0.000213	0.006761	6.93E-05	0.000302
GO_NEGATIVE_REGULATION_OF_PRODUCTION_OF_MOLECULAR_MEDIATOR_OF_IMMUNE_RESPONSE	28	Up	0.000213	0.006761	0.000129	0.000302
GO_NEUTRAL_AMINO_ACID_TRANSPORT	21	Up	0.000215	0.006761	0.000366	0.000416
GO_REGULATION_OF_MICROTUBULE-BASED_MOVEMENT	18	Down	0.000215	0.006761	1.4E-05	0.000302
GO_REGULATION_OF_B_CELL_PROLIFERATION	60	Up	0.000222	0.006761	0.000169	0.000302
GO_SMOOTHENED_SIGNALING_PATHWAY	118	Down	0.000222	0.006761	0.000148	0.000302
GO_REGULATION_OF_ATPASE_ACTIVITY	47	Down	0.000225	0.006761	0.000199	0.000307
GO_POSITIVE_REGULATION_OF_CD4-POSITIVE,_ALPHA-BETA_T_CELL_DIFFERENTIATION	23	Up	0.000226	0.006761	0.0001	0.000302
GO_RESPONSE_TO_ISCHEMIA	20	Up	0.000226	0.006761	6.53E-05	0.000302
GO_MONONUCLEAR_CELL_MIGRATION	61	Up	0.000227	0.006761	6.73E-05	0.000302
GO_REGULATION_OF_MAST_CELL_ACTIVATION	43	Up	0.000229	0.006761	0.000179	0.000302
GO_RESPONSE_TO_PEPTIDOGLYCAN	13	Up	0.000234	0.006761	4.8E-05	0.000302
GO_PLATELET_MORPHOGENESIS	17	Up	0.000234	0.006761	0.000264	0.00034
GO_REGULATION_OF_ACUTE_INFLAMMATORY_RESPONSE_TO_ANTIGENIC_STIMULUS	12	Up	0.000234	0.006761	0.000195	0.000306
GO_APOPTOTIC_CELL_CLEARANCE	35	Up	0.000237	0.006761	6.61E-05	0.000302
GO_NATURAL_KILLER_CELL_ACTIVATION_INVOLVED_IN_IMMUNE_RESPONSE	11	Up	0.00024	0.006761	0.000135	0.000302
GO_NEGATIVE_REGULATION_OF_TYPE_2_IMMUNE_RESPONSE	13	Up	0.00024	0.006761	0.000167	0.000302
GO_REGULATION_OF_CELLULAR_RESPONSE_TO_DRUG	31	Up	0.000242	0.006761	0.000117	0.000302
GO_OOGENESIS	52	Down	0.000243	0.006761	0.00012	0.000302
GO_MICROTUBULE_BUNDLE_FORMATION	59	Down	0.000243	0.006761	0.000168	0.000302
GO_REGULATION_OF_MHC_CLASS_II_BIOSYNTHETIC_PROCESS	12	Up	0.000245	0.006761	7.05E-05	0.000302
GO_REGULATION_OF_DNA-DEPENDENT_DNA_REPLICATION	44	Down	0.000245	0.006761	0.000323	0.000382
GO_MAST_CELL_ACTIVATION_INVOLVED_IN_IMMUNE_RESPONSE	43	Up	0.000248	0.006761	0.000182	0.000302
GO_MAST_CELL_DEGRANULATION	43	Up	0.000248	0.006761	0.000182	0.000302
GO_MHC_CLASS_II_BIOSYNTHETIC_PROCESS	13	Up	0.000253	0.006761	7.47E-05	0.000302
GO_LYSOSOME_LOCALIZATION	64	Up	0.000253	0.006761	0.000218	0.000316
GO_NEGATIVE_REGULATION_OF_NEUROLOGICAL_SYSTEM_PROCESS	13	Up	0.000259	0.006761	1.51E-05	0.000302
GO_VENTRICULAR_SYSTEM_DEVELOPMENT	26	Down	0.000261	0.006761	0.000124	0.000302
GO_RENAL_FILTRATION_CELL_DIFFERENTIATION	14	Up	0.000264	0.006761	1.92E-05	0.000302
GO_GLOMERULAR_VISCERAL_EPITHELIAL_CELL_DIFFERENTIATION	14	Up	0.000264	0.006761	1.92E-05	0.000302
GO_GLOMERULAR_EPITHELIAL_CELL_DIFFERENTIATION	14	Up	0.000264	0.006761	1.92E-05	0.000302
GO_NEGATIVE_REGULATION_OF_AXON_REGENERATION	13	Down	0.000267	0.006761	0.000113	0.000302
GO_POSITIVE_REGULATION_OF_ALPHA-BETA_T_CELL_DIFFERENTIATION	37	Up	0.000267	0.006761	0.000111	0.000302
GO_NEGATIVE_REGULATION_OF_ENDOPLASMIC_RETICULUM_STRESS-INDUCED_INTRINSIC_APOPTOTIC_SIGNALING_PATHWAY	16	Up	0.000268	0.006761	0.000121	0.000302
GO_NEGATIVE_REGULATION_OF_RESPONSE_TO_REACTIVE_OXYGEN_SPECIES	21	Up	0.000268	0.006761	5.79E-05	0.000302
GO_NEGATIVE_REGULATION_OF_HYDROGEN_PEROXIDE-INDUCED_CELL_DEATH	21	Up	0.000268	0.006761	5.79E-05	0.000302
GO_NEGATIVE_REGULATION_OF_CELLULAR_RESPONSE_TO_DRUG	21	Up	0.000268	0.006761	5.79E-05	0.000302
GO_REGULATION_OF_MONOCYTE_DIFFERENTIATION	14	Up	0.000271	0.006761	0.000372	0.000421
GO_MAST_CELL_MEDIATED_IMMUNITY	44	Up	0.000274	0.006761	0.0002	0.000307
GO_POSITIVE_REGULATION_OF_CALCIUM-MEDIATED_SIGNALING	37	Up	0.000276	0.006761	0.000132	0.000302
GO_POSITIVE_REGULATION_OF_MYOBLAST_FUSION	14	Up	0.000276	0.006761	0.000228	0.000322
GO_NON-MOTILE_CILIUM_ASSEMBLY	60	Down	0.00028	0.006761	6.04E-05	0.000302
GO_REGULATION_OF_FIBROBLAST_MIGRATION	33	Up	0.000281	0.006761	0.000304	0.000369
GO_REGULATION_OF_MAST_CELL_ACTIVATION_INVOLVED_IN_IMMUNE_RESPONSE	31	Up	0.000282	0.006761	0.000162	0.000302
GO_REGULATION_OF_MAST_CELL_DEGRANULATION	31	Up	0.000282	0.006761	0.000162	0.000302
GO_NEGATIVE_REGULATION_OF_INTRACELLULAR_ESTROGEN_RECEPTOR_SIGNALING_PATHWAY	11	Down	0.000284	0.006761	0.000385	0.000432
GO_SIGNAL_TRANSDUCTION_IN_ABSENCE_OF_LIGAND	72	Up	0.000288	0.006761	0.000347	0.0004
GO_EXTRINSIC_APOPTOTIC_SIGNALING_PATHWAY_IN_ABSENCE_OF_LIGAND	72	Up	0.000288	0.006761	0.000347	0.0004
GO_EPITHELIAL_STRUCTURE_MAINTENANCE	17	Up	0.000289	0.006761	0.000377	0.000425
GO_MAST_CELL_ACTIVATION	61	Up	0.00029	0.006761	0.0002	0.000307
GO_REGULATION_OF_ATP_BIOSYNTHETIC_PROCESS	37	Up	0.000291	0.006761	0.000148	0.000302
GO_MYELOID_DENDRITIC_CELL_DIFFERENTIATION	20	Up	0.000294	0.006761	4.66E-05	0.000302
GO_INTRACILIARY_TRANSPORT	38	Down	0.000295	0.006761	0.000199	0.000307
GO_LEUKOCYTE_HOMEOSTASIS	97	Up	0.000295	0.006761	9.93E-05	0.000302
GO_MUCOSAL-ASSOCIATED_LYMPHOID_TISSUE_DEVELOPMENT	13	Up	0.000297	0.006761	0.000319	0.000379
GO_PEYER'S_PATCH_DEVELOPMENT	13	Up	0.000297	0.006761	0.000319	0.000379
GO_REGULATION_OF_GTP_BINDING	18	Down	0.000299	0.006761	0.000262	0.000338

GO_NEGATIVE_REGULATION_OF_PEPTIDE_SECRETION	108	Up	0.0003	0.006761	9.9E-05	0.000302
GO_SENSORY_PERCEPTION_OF_TEMPERATURE_STIMULUS	14	Up	0.0003	0.006761	0.000473	0.00051
GO_B_CELL_DIFFERENTIATION	98	Up	0.000302	0.006761	0.000139	0.000302
GO_LAYER_FORMATION_IN_CEREBRAL_CORTEX	11	Down	0.000303	0.006761	0.000156	0.000302
GO_CENTROMERE_COMPLEX_ASSEMBLY	26	Down	0.000305	0.006761	0.00019	0.000305
GO_ACUTE_INFLAMMATORY_RESPONSE_TO_ANTIGENIC_STIMULUS	20	Up	0.000306	0.006761	0.000118	0.000302
GO_MONOCYTE_CHEMOTAXIS	43	Up	0.000313	0.006761	5.63E-05	0.000302
GO_OSTEOCLAST_DIFFERENTIATION	91	Up	0.000313	0.006761	0.000135	0.000302
GO_BASIC_AMINO_ACID_TRANSPORT	11	Up	0.000313	0.006761	0.000392	0.000438
GO_REGULATION_OF_SMOOTHENED_SIGNALING_PATHWAY	71	Down	0.000315	0.006761	0.000239	0.000327
GO_MANNOSYLATION	16	Down	0.000318	0.006761	0.00017	0.000302
GO_ANTIGEN_PROCESSING_AND_PRESENTATION_OF_PEPTIDE_OR_POLYSACCHARIDE_ANTIGEN_VIA_MHC_CLASS_II	20	Up	0.000318	0.006761	0.000159	0.000302
GO_POSITIVE_REGULATION_OF_SYNCYTIUM_FORMATION_BY_PLASMA_MEMBRANE_FUSION	19	Up	0.000319	0.006761	0.000276	0.000348
GO_PLASMA_LIPOPROTEIN_PARTICLE_ASSEMBLY	14	Up	0.000319	0.006761	3.52E-05	0.000302
GO_LEUKOCYTE_DEGRANULATION	63	Up	0.00032	0.006761	0.000186	0.000303
GO_MYELOID_LEUKOCYTE_DIFFERENTIATION	188	Up	0.000321	0.006761	0.000122	0.000302
GO_REGULATION_OF_RESPIRATORY_GASEOUS_EXCHANGE	14	Down	0.000322	0.006761	0.000227	0.000321
GO_POSITIVE_REGULATION_OF_MACROPHAGE_CHEMOTAXIS	16	Up	0.000324	0.006761	8.45E-05	0.000302
GO_POSITIVE_REGULATION_OF_FIBROBLAST_MIGRATION	16	Up	0.000326	0.006761	0.000525	0.000556
GO_MONOCYTE_DIFFERENTIATION	26	Up	0.000326	0.006761	0.000142	0.000302
GO_MONONUCLEAR_CELL_DIFFERENTIATION	26	Up	0.000326	0.006761	0.000142	0.000302
GO_REGULATION_OF_MONONUCLEAR_CELL_MIGRATION	40	Up	0.000328	0.006761	6.33E-05	0.000302
GO_POSITIVE_REGULATION_OF_LEUKOCYTE_DIFFERENTIATION	139	Up	0.000329	0.006761	0.000131	0.000302
GO_SIGNAL_TRANSDUCTION_INVOLVED_IN_G2_DNA_DAMAGE_CHECKPOINT	13	Down	0.00033	0.006761	0.000209	0.000311
GO_POSITIVE_REGULATION_OF_DNA_REPAIR	50	Down	0.00033	0.006761	0.000245	0.000329
GO_CELLULAR_RESPONSE_TO_ANGIOTENSIN	14	Up	0.000333	0.006761	0.000171	0.000302
GO_RESPONSE_TO_ANGIOTENSIN	14	Up	0.000333	0.006761	0.000171	0.000302
GO_INTERLEUKIN-6_SECRETION	34	Up	0.000334	0.006761	0.00011	0.000302
GO_TUMOR_NECROSIS_FACTOR-MEDIATED_SIGNALING_PATHWAY	66	Up	0.000335	0.006761	0.00016	0.000302
GO_NEGATIVE_REGULATION_OF_HORMONE_SECRETION	61	Up	0.000337	0.006761	7.49E-05	0.000302
GO_CELLULAR_RESPONSE_TO_TUMOR_NECROSIS_FACTOR	134	Up	0.000337	0.006761	0.000129	0.000302
GO_SCF-DEPENDENT_PROTEASOMAL_UBIQUITIN-DEPENDENT_PROTEIN_CATABOLIC_PROCESS	23	Down	0.000342	0.006761	0.000226	0.000321
GO_CELLULAR_BIOGENIC_AMINE_CATABOLIC_PROCESS	11	Up	0.000342	0.006761	0.000359	0.000409
GO_INTRACILIARY_RETROGRADE_TRANSPORT	12	Down	0.000342	0.006761	0.000451	0.00049
GO_REGULATION_OF_LEUKOCYTE_APOPTOTIC_PROCESS	89	Up	0.000345	0.006761	0.000104	0.000302
GO_NEGATIVE_REGULATION_OF_ORGANELLE_ASSEMBLY	29	Down	0.000348	0.006761	0.000159	0.000302
GO_ARP2/3_COMPLEX-MEDIATED_ACTIN_NUCLEATION	31	Up	0.00035	0.006761	0.000111	0.000302
GO_LIPOPOLYSACCHARIDE-MEDIATED_SIGNALING_PATHWAY	45	Up	0.000351	0.006761	6.12E-05	0.000302
GO_MATURE_B_CELL_DIFFERENTIATION_INVOLVED_IN_IMMUNE_RESPONSE	18	Up	0.000351	0.006761	0.000138	0.000302
GO_MATURE_B_CELL_DIFFERENTIATION	23	Up	0.000353	0.006761	0.00016	0.000302
GO_LEUKOCYTE_APOPTOTIC_PROCESS	107	Up	0.000356	0.006761	0.000122	0.000302
GO_T_CELL_CYTOKINE_PRODUCTION	32	Up	0.000357	0.006761	0.000113	0.000302
GO_AMINOGLYCAN_BIOSYNTHETIC_PROCESS	45	Up	0.000359	0.006761	7.14E-05	0.000302
GO_REGULATION_OF_EXIT_FROM_MITOSIS	12	Down	0.00036	0.006761	0.000367	0.000417
GO_NEGATIVE_REGULATION_OF_CYSTEINE-TYPE_ENDOPEPTIDASE_ACTIVITY	83	Up	0.00036	0.006761	6.61E-05	0.000302
GO_NEGATIVE_REGULATION_OF_INTERLEUKIN-6_PRODUCTION	36	Up	0.00036	0.006761	0.000197	0.000307
GO_REGULATION_OF_CILIUM_ASSEMBLY	43	Down	0.000361	0.006761	6.5E-05	0.000302
GO_POSITIVE_REGULATION_OF_HISTONE_H3-K4_METHYLATION	14	Down	0.000363	0.006761	0.000148	0.000302
GO_REGULATION_OF_ENDOPLASMIC_RETICULUM_STRESS-INDUCED_INTRINSIC_APOPTOTIC_SIGNALING_PATHWAY	25	Up	0.000365	0.006761	0.00017	0.000302
GO_REGULATION_OF_ALPHA-BETA_T_CELL_DIFFERENTIATION	50	Up	0.000365	0.006761	1E-04	0.000302
GO_PLASMA_MEMBRANE_INVAGINATION	53	Up	0.000367	0.006761	0.000147	0.000302
GO_GLUTAMATE_METABOLIC_PROCESS	21	Up	0.000367	0.006761	0.000153	0.000302
GO_ACTIVATED_T_CELL_PROLIFERATION	41	Up	0.000368	0.006761	0.000137	0.000302
GO_POSITIVE_REGULATION_OF_REGULATED_SECRETORY_PATHWAY	42	Up	0.00037	0.006761	0.000263	0.000339
GO_ARF_PROTEIN_SIGNAL_TRANSDUCTION	38	Up	0.000373	0.006761	7.79E-05	0.000302
GO_REGULATION_OF_ARF_PROTEIN_SIGNAL_TRANSDUCTION	38	Up	0.000373	0.006761	7.79E-05	0.000302
GO_POSITIVE_REGULATION_OF_LIPID_STORAGE	15	Up	0.000373	0.006761	0.000143	0.000302
GO_SYNAPSE_MATURATION	16	Down	0.000374	0.006761	0.000296	0.000363
GO_POSITIVE_REGULATION_OF_CD4-POSITIVE,_ALPHA-BETA_T_CELL_ACTIVATION	27	Up	0.000376	0.006761	0.000146	0.000302
GO_REGULATION_OF_CELLULAR_PH	63	Up	0.000377	0.006761	9.96E-05	0.000302
GO_PHAGOCYTOSIS	161	Up	0.000378	0.006761	0.00017	0.000302
GO_PHAGOCYTOSIS,_ENGULFMENT	44	Up	0.000378	0.006761	0.000162	0.000302
GO_POSITIVE_REGULATION_OF_T_CELL_DIFFERENTIATION	74	Up	0.000378	0.006761	0.000143	0.000302
GO_MYELOID_DENDRITIC_CELL_ACTIVATION	32	Up	0.000381	0.006761	8.51E-05	0.000302
GO_AMINE_CATABOLIC_PROCESS	12	Up	0.000382	0.006761	0.000389	0.000435
GO_CD4-POSITIVE,_ALPHA-BETA_T_CELL_DIFFERENTIATION	64	Up	0.000383	0.006761	0.000119	0.000302
GO_MAINTENANCE_OF_LOCATION_IN_CELL	78	Down	0.000388	0.006761	7.97E-05	0.000302
GO_REGULATION_OF_PROTEIN_SECRETION	353	Up	0.000389	0.006761	0.000141	0.000302
GO_CENTRIOLE_REPLICATION	29	Down	0.00039	0.006761	0.000213	0.000313
GO_CENTRIOLE_ASSEMBLY	29	Down	0.00039	0.006761	0.000213	0.000313
GO_MYELOID_CELL_DIFFERENTIATION	331	Up	0.00039	0.006761	0.000115	0.000302
GO_POSITIVE_REGULATION_OF_MYOTUBE_DIFFERENTIATION	23	Up	0.000392	0.006761	0.00021	0.000311
GO_POSITIVE_REGULATION_OF_ACUTE_INFLAMMATORY_RESPONSE	24	Up	0.000395	0.006761	0.000268	0.000342
GO_EPITHELIAL_CELL-CELL_ADHESION	13	Up	0.000395	0.006761	2.28E-05	0.000302
GO_INTRINSIC_APOPTOTIC_SIGNALING_PATHWAY_IN_RESPONSE_TO_OXIDATIVE_STRESS	39	Down	0.000395	0.006761	0.000151	0.000302
GO_MYELOID_CELL_ACTIVATION_INVOLVED_IN_IMMUNE_RESPONSE	74	Up	0.000401	0.006761	0.000178	0.000302
GO_REGULATION_OF_CELL_CYCLE_PHASE_TRANSITION	256	Down	0.000401	0.006761	0.000205	0.000309
GO_POSITIVE_REGULATION_OF_LIPID_LOCALIZATION	61	Up	0.000401	0.006761	0.000134	0.000302
GO_CHLORIDE_ION_HOMEOSTASIS	14	Up	0.000401	0.006761	0.000325	0.000384
GO_POSITIVE_REGULATION_OF_ATPASE_ACTIVITY	32	Down	0.000401	0.006761	0.000143	0.000302
GO_T-HELPER_1_CELL_DIFFERENTIATION	20	Up	0.000402	0.006761	5.86E-05	0.000302
GO_OOCYTE_DEVELOPMENT	30	Down	0.000403	0.006761	0.000233	0.000324
GO_REGULATION_OF_LEUKOCYTE_DIFFERENTIATION	243	Up	0.000404	0.006761	0.000157	0.000302
GO_LEUKOCYTE_DIFFERENTIATION	456	Up	0.000404	0.006761	0.000143	0.000302
GO_MEMBRANE_INVAGINATION	60	Up	0.000406	0.006761	0.000144	0.000302
GO_CYTOKINE_PRODUCTION_INVOLVED_IN_IMMUNE_RESPONSE	79	Up	0.000407	0.006761	0.000128	0.000302
GO_PROTEIN_SECRETION	439	Up	0.000408	0.006761	0.000149	0.000302
GO_INTERLEUKIN-10_PRODUCTION	45	Up	0.000411	0.006761	0.000211	0.000312
GO_INTERLEUKIN-4_PRODUCTION	31	Up	0.000412	0.006761	0.000306	0.00037
GO_POSITIVE_REGULATION_OF_LIPID_TRANSPORT	47	Up	0.000413	0.006761	0.000124	0.000302
GO_REGULATION_OF_INFLAMMATORY_RESPONSE_TO_ANTIGENIC_STIMULUS	22	Up	0.000415	0.006761	0.000162	0.000302
GO_SNRNA_PROCESSING	24	Down	0.000416	0.006761	0.000123	0.000302
GO_REGULATION_OF_MITOTIC_CELL_CYCLE_PHASE_TRANSITION	230	Down	0.000416	0.006761	0.000222	0.000319
GO_REGULATION_OF_MUCUS_SECRETION	11	Up	0.000416	0.006761	9.48E-05	0.000302
GO_ANTIGEN_PROCESSING_AND_PRESENTATION_OF_PEPTIDE_ANTIGEN_VIA_MHC_CLASS_II	19	Up	0.000416	0.006761	0.000184	0.000303
GO_NEGATIVE_REGULATION_OF_CYSTEINE-TYPE_ENDOPEPTIDASE_ACTIVITY_INVOLVED_IN_APOPTOTIC_PROCESS	76	Up	0.000418	0.006761	6.82E-05	0.000302
GO_NEGATIVE_REGULATION_OF_FIBROBLAST_PROLIFERATION	33	Up	0.000419	0.006761	8.02E-05	0.000302
GO_REGULATION_OF_LEUKOCYTE_DEGRANULATION	44	Up	0.000422	0.006761	0.0002	0.000307
GO_CIRCADIAN_SLEEP/WAKE_CYCLE_PROCESS	13	Up	0.000424	0.006761	0.00021	0.000311
GO_CIRCADIAN_SLEEP/WAKE_CYCLE,_SLEEP	13	Up	0.000424	0.006761	0.00021	0.000311
GO_EMBRYONIC_DIGESTIVE_TRACT_DEVELOPMENT	25	Up	0.000426	0.006761	0.000105	0.000302
GO_MITOTIC_RECOMBINATION	24	Down	0.000427	0.006761	0.0003	0.000366
GO_POSITIVE_REGULATION_OF_G2/M_TRANSITION_OF_MITOTIC_CELL_CYCLE	21	Down	0.000428	0.006761	0.000247	0.00033
GO_CELL_CYCLE_G1/S_PHASE_TRANSITION	175	Down	0.000428	0.006761	0.000194	0.000306
GO_GERMINAL_CENTER_FORMATION	14	Up	0.000432	0.006761	4.49E-05	0.000302
GO_POSITIVE_REGULATION_OF_CELL_CYCLE_PHASE_TRANSITION	74	Down	0.000432	0.006761	0.000256	0.000335
GO_KINETOCHORE_ASSEMBLY	14	Down	0.000433	0.006761	0.000197	0.000307
GO_REGULATION_OF_T-HELPER_1_CELL_DIFFERENTIATION	11	Up	0.000436	0.006761	4.19E-05	0.000302
GO_MITOTIC_CYTOKINESIS	63	Down	0.000436	0.006761	0.000218	0.000316
GO_SNRNA_METABOLIC_PROCESS	40	Down	0.000437	0.006761	9.35E-05	0.000302
GO_ORGANELLE_ASSEMBLY	621	Down	0.000437	0.006761	0.000139	0.000302
GO_REGULATION_OF_CELL_ADHESION_MEDIATED_BY_INTEGRIN	32	Up	0.000437	0.006761	0.000148	0.000302
GO_POSITIVE_REGULATION_OF_LYMPHOCYTE_DIFFERENTIATION	86	Up	0.000439	0.006761	0.000143	0.000302
GO_LIPID_PHOSPHORYLATION	34	Up	0.000439	0.006761	0.000121	0.000302
GO_REGULATION_OF_MYELOID_LEUKOCYTE_MEDIATED_IMMUNITY	50	Up	0.000442	0.006761	0.000195	0.000306
GO_REGULATION_OF_MYELOID_LEUKOCYTE_DIFFERENTIATION	108	Up	0.000443	0.006761	0.000147	0.000302
GO_REGULATION_OF_LYMPHOCYTE_APOPTOTIC_PROCESS	61	Up	0.000444	0.006761	8.12E-05	0.000302
GO_NEGATIVE_REGULATION_OF_NF-KAPPAB_TRANSCRIPTION_FACTOR_ACTIVITY	71	Up	0.000444	0.006761	0.000103	0.000302
GO_MULTI-ORGANISM_CELLULAR_PROCESS	30	Up	0.000446	0.006761	5.85E-05	0.000302
GO_L-AMINO_ACID_TRANSPORT	39	Up	0.000446	0.006761	0.000239	0.000327
GO_REGULATION_OF_ANTIGEN_PROCESSING_AND_PRESENTATION	15	Up	0.000447	0.006761	0.000165	0.000302
GO_NEGATIVE_REGULATION_OF_LEUKOCYTE_APOPTOTIC_PROCESS	51	Up	0.000452	0.006761	0.000127	0.000302
GO_RESPONSE_TO_UV-C	13	Down	0.000453	0.006761	9.26E-05	0.000302
GO_POSITIVE_REGULATION_OF_CELL_CYCLE_G2/M_PHASE_TRANSITION	25	Down	0.000455	0.006761	0.000255	0.000335
GO_POSITIVE_REGULATION_OF_CYTOKINESIS	24	Down	0.000455	0.006761	0.000266	0.000341
GO_RNA_PHOSPHODIESTER_BOND_HYDROLYSIS,_EXONUCLEOLYTIC	13	Down	0.000458	0.006761	0.000197	0.000307
GO_CELLULAR_RESPONSE_TO_MOLECULE_OF_BACTERIAL_ORIGIN	146	Up	0.000462	0.006761	0.000113	0.000302

GO_REGULATION_OF_INTERLEUKIN-4_PRODUCTION	24	Up	0.000463	0.006761	0.000369	0.000418
GO_NEGATIVE_REGULATION_OF_PROTEIN_TRANSPORT	149	Up	0.000463	0.006761	9.1E-05	0.000302
GO_MICROTUBULE-BASED_MOVEMENT	198	Down	0.000464	0.006761	0.000177	0.000302
GO_POSITIVE_REGULATION_OF_ATP_BIOSYNTHETIC_PROCESS	19	Up	0.000466	0.006761	0.000563	0.000592
GO_REGULATION_OF_MYOBLAST_FUSION	17	Up	0.000466	0.006761	0.000219	0.000317
GO_POSITIVE_REGULATION_OF_MYELOID_LEUKOCYTE_CYTOKINE_PRODUCTION_INVOLVED_IN_IMMUNE_RESPONSE	11	Up	0.000467	0.006761	0.000143	0.000302
GO_CELLULAR_RESPONSE_TO_BIOTIC_STIMULUS	164	Up	0.000468	0.006761	0.000109	0.000302
GO_PHOSPHATIDYLINOSITOL_PHOSPHORYLATION	27	Up	0.00047	0.006761	0.000155	0.000302
GO_CELLULAR_RESPONSE_TO_LIPOPOLYSACCHARIDE	137	Up	0.000471	0.006761	0.000106	0.000302
GO_POSITIVE_REGULATION_OF_GENE_EXPRESSION,_EPIGENETIC	19	Down	0.000471	0.006761	0.000307	0.000371
GO_MUCUS_SECRETION	12	Up	0.000475	0.006761	0.000121	0.000302
GO_REGULATION_OF_I-KAPPAB_KINASE/NF-KAPPAB_SIGNALING	140	Up	0.000475	0.006761	0.000187	0.000304
GO_REGULATION_OF_INTRACELLULAR_PH	59	Up	0.000482	0.006761	0.000106	0.000302
GO_POSITIVE_REGULATION_OF_RESPONSE_TO_DNA_DAMAGE_STIMULUS	81	Down	0.000482	0.006761	0.000213	0.000313
GO_CELL_CYCLE_PHASE_TRANSITION	348	Down	0.000483	0.006761	0.000188	0.000305
GO_REGULATION_OF_INTERLEUKIN-10_PRODUCTION	42	Up	0.000484	0.006761	0.000207	0.00031
GO_CELL_ACTIVATION_INVOLVED_IN_IMMUNE_RESPONSE	211	Up	0.000489	0.006761	0.00013	0.000302
GO_PROTEIN_LOCALIZATION_TO_CILIUM	33	Down	0.00049	0.006761	0.000258	0.000336
GO_NEGATIVE_REGULATION_OF_MITOTIC_CELL_CYCLE	200	Down	0.000491	0.006761	0.000183	0.000303
GO_PROTEIN-LIPID_COMPLEX_ASSEMBLY	16	Up	0.000492	0.006761	3.48E-05	0.000302
GO_MITOTIC_CELL_CYCLE_PHASE_TRANSITION	317	Down	0.000493	0.006761	0.000196	0.000306
GO_INTRACELLULAR_PH_REDUCTION	33	Up	0.000494	0.006761	0.000127	0.000302
GO_VITAMIN_BIOSYNTHETIC_PROCESS	18	Up	0.000498	0.006761	0.000328	0.000386
GO_CELLULAR_RESPONSE_TO_LIGHT_STIMULUS	77	Down	0.000498	0.006761	0.000201	0.000307
GO_POSITIVE_REGULATION_OF_ICOSANOID_SECRETION	13	Up	0.000499	0.006761	0.000212	0.000313
GO_POSITIVE_REGULATION_OF_PROTEIN_SECRETION	224	Up	0.0005	0.006761	0.000184	0.000303
GO_POSITIVE_REGULATION_OF_PROTEIN_DEACETYLATION	26	Up	0.0005	0.006761	2.87E-05	0.000302
GO_LEUKOCYTE_CHEMOTAXIS	173	Up	0.0005	0.006761	0.000108	0.000302
GO_MYELOID_LEUKOCYTE_ACTIVATION	167	Up	0.000502	0.006761	0.000129	0.000302
GO_DENDRITIC_CELL_DIFFERENTIATION	35	Up	0.000502	0.006761	9.14E-05	0.000302
GO_POSITIVE_REGULATION_OF_INFLAMMATORY_RESPONSE_TO_ANTIGENIC_STIMULUS	12	Up	0.000502	0.006761	0.000222	0.000319
GO_NEGATIVE_REGULATION_OF_ATP_BIOSYNTHETIC_PROCESS	13	Up	0.000503	0.006761	0.000218	0.000316
GO_FIBROBLAST_ACTIVATION	11	Up	0.000503	0.006761	0.000172	0.000302
GO_REGULATION_OF_INTRACELLULAR_ESTROGEN_RECEPTOR_SIGNALING_PATHWAY	29	Down	0.000503	0.006761	0.000404	0.000448
GO_REGULATION_OF_CIRCADIAN_SLEEP/WAKE_CYCLE	12	Up	0.000506	0.006761	0.000237	0.000325
GO_REGULATION_OF_CIRCADIAN_SLEEP/WAKE_CYCLE,_SLEEP	12	Up	0.000506	0.006761	0.000237	0.000325
GO_PLATELET-DERIVED_GROWTH_FACTOR_RECEPTOR-BETA_SIGNALING_PATHWAY	11	Up	0.000508	0.006761	0.000161	0.000302
GO_ACTIVATION-INDUCED_CELL_DEATH_OF_T_CELLS	15	Up	0.000508	0.006761	9.48E-05	0.000302
GO_REGULATION_OF_DNA_DAMAGE_CHECKPOINT	17	Down	0.000508	0.006761	5.37E-05	0.000302
GO_AMP_METABOLIC_PROCESS	12	Down	0.00051	0.006761	2.19E-05	0.000302
GO_RESPONSE_TO_MOLECULE_OF_BACTERIAL_ORIGIN	251	Up	0.000511	0.006761	9.45E-05	0.000302
GO_I-KAPPAB_KINASE/NF-KAPPAB_SIGNALING	169	Up	0.000514	0.006761	0.000172	0.000302
GO_REGULATION_OF_MACROPHAGE_DIFFERENTIATION	19	Up	0.000515	0.006761	7.06E-05	0.000302
GO_POSITIVE_REGULATION_OF_PHAGOCYTOSIS	53	Up	0.000515	0.006761	0.000173	0.000302
GO_G2/M_TRANSITION_OF_MITOTIC_CELL_CYCLE	99	Down	0.000516	0.006761	0.000207	0.00031
GO_CELL_CYCLE_G2/M_PHASE_TRANSITION	115	Down	0.000517	0.006761	0.00019	0.000305
GO_PLASMA_MEMBRANE_BOUNDED_CELL_PROJECTION_ASSEMBLY	397	Down	0.000518	0.006761	0.000184	0.000303
GO_GLOMERULAR_EPITHELIUM_DEVELOPMENT	16	Up	0.00052	0.006761	3.75E-05	0.000302
GO_RESPONSE_TO_RADIATION	317	Down	0.00052	0.006761	0.000147	0.000302
GO_NEGATIVE_REGULATION_OF_CELL_CYCLE_PHASE_TRANSITION	142	Down	0.000522	0.006761	0.000181	0.000302
GO_NEGATIVE_REGULATION_OF_MITOTIC_CELL_CYCLE_PHASE_TRANSITION	127	Down	0.000522	0.006761	0.00019	0.000306
GO_CELL_CYCLE_DNA_REPLICATION	36	Down	0.000525	0.006761	0.000254	0.000333
GO_NEGATIVE_REGULATION_OF_INFLAMMATORY_RESPONSE	100	Up	0.000525	0.006761	0.000122	0.000302
GO_PROTEIN-DNA_COMPLEX_ASSEMBLY	123	Down	0.000527	0.006761	0.000337	0.000393
GO_LEUKOCYTE_ACTIVATION_INVOLVED_IN_IMMUNE_RESPONSE	207	Up	0.000528	0.006761	0.000129	0.000302
GO_CALCIUM_ION_TRANSPORT	268	Up	0.00053	0.006761	0.000119	0.000302
GO_CORPUS_CALLOSUM_DEVELOPMENT	17	Down	0.00053	0.006761	5.61E-05	0.000302
GO_RESPONSE_TO_TUMOR_NECROSIS_FACTOR	150	Up	0.000532	0.006761	0.000111	0.000302
GO_REGULATION_OF_PRODUCTION_OF_MOLECULAR_MEDIATOR_OF_IMMUNE_RESPONSE	112	Up	0.000533	0.006761	0.000111	0.000302
GO_INFLAMMATORY_RESPONSE	515	Up	0.000536	0.006761	0.000143	0.000302
GO_NEGATIVE_REGULATION_OF_ERYTHROCYTE_DIFFERENTIATION	11	Up	0.000537	0.006761	0.000112	0.000302
GO_NATURAL_KILLER_CELL_DIFFERENTIATION	26	Up	0.000538	0.006761	0.000223	0.000319
GO_CENTROSOME_CYCLE	114	Down	0.000538	0.006761	0.000179	0.000302
GO_VACUOLAR_ACIDIFICATION	19	Up	0.000538	0.006761	0.000131	0.000302
GO_MICROTUBULE_ORGANIZING_CENTER_ORGANIZATION	120	Down	0.000538	0.006761	0.000177	0.000302
GO_POSITIVE_REGULATION_OF_LEUKOCYTE_DEGRANULATION	25	Up	0.000541	0.006761	0.000217	0.000315
GO_REGULATION_OF_SYNCYTIUM_FORMATION_BY_PLASMA_MEMBRANE_FUSION	24	Up	0.000541	0.006761	0.000268	0.000341
GO_MUCOPOLYSACCHARIDE_METABOLIC_PROCESS	50	Up	0.000542	0.006761	0.000116	0.000302
GO_REGULATION_OF_STEROL_TRANSPORT	28	Up	0.000543	0.006761	0.000141	0.000302
GO_REGULATION_OF_CHOLESTEROL_TRANSPORT	28	Up	0.000543	0.006761	0.000141	0.000302
GO_CELLULAR_RESPONSE_TO_AMMONIUM_ION	24	Up	0.000544	0.006761	0.000153	0.000302
GO_POSITIVE_REGULATION_OF_MYELOID_LEUKOCYTE_DIFFERENTIATION	55	Up	0.000546	0.006761	0.000128	0.000302
GO_MAINTENANCE_OF_PROTEIN_LOCATION_IN_CELL	63	Down	0.000547	0.006761	4.94E-05	0.000302
GO_REGULATION_OF_POSTSYNAPTIC_MEMBRANE_ORGANIZATION	11	Up	0.000551	0.006761	0.000162	0.000302
GO_POSITIVE_REGULATION_OF_BONE_RESORPTION	21	Up	0.000553	0.006761	0.000144	0.000302
GO_POSITIVE_REGULATION_OF_BONE_REMODELING	21	Up	0.000553	0.006761	0.000144	0.000302
GO_NEGATIVE_REGULATION_OF_ESTABLISHMENT_OF_PROTEIN_LOCALIZATION	153	Up	0.000553	0.006761	8.96E-05	0.000302
GO_REGULATION_OF_REGULATED_SECRETORY_PATHWAY	99	Up	0.000554	0.006761	0.000212	0.000313
GO_NEGATIVE_REGULATION_OF_IMMUNE_SYSTEM_PROCESS	374	Up	0.000554	0.006761	0.000151	0.000302
GO_REGULATION_OF_MYOBLAST_DIFFERENTIATION	44	Up	0.000555	0.006761	0.000151	0.000302
GO_LYMPHOCYTE_CHEMOTAXIS	41	Up	0.000557	0.006761	8.02E-05	0.000302
GO_RESPIRATORY_BURST	22	Up	0.000557	0.006761	0.000178	0.000302
GO_ACUTE_INFLAMMATORY_RESPONSE	88	Up	0.00056	0.006761	0.000187	0.000304
GO_TELOMERE_MAINTENANCE_VIA_RECOMBINATION	16	Down	0.000562	0.006761	0.000297	0.000363
GO_CELLULAR_MONOVALENT_INORGANIC_CATION_HOMEOSTASIS	76	Up	0.000562	0.006761	0.000123	0.000302
GO_PHOSPHOLIPID_TRANSPORT	46	Up	0.000563	0.006761	0.000126	0.000302
GO_CALCIUM_ION_IMPORT	50	Up	0.000564	0.006761	4.7E-05	0.000302
GO_NEGATIVE_REGULATION_OF_IMMUNE_EFFECTOR_PROCESS	104	Up	0.000565	0.006761	0.000104	0.000302
GO_REGULATION_OF_ACUTE_INFLAMMATORY_RESPONSE	48	Up	0.000565	0.006761	0.000237	0.000326
GO_CELLULAR_RESPONSE_TO_RADIATION	128	Down	0.000571	0.006761	0.000164	0.000302
GO_SIGNAL_TRANSDUCTION_INVOLVED_IN_CELL_CYCLE_CHECKPOINT	28	Down	0.000573	0.006761	8.37E-05	0.000302
GO_SIGNAL_TRANSDUCTION_INVOLVED_IN_DNA_INTEGRITY_CHECKPOINT	28	Down	0.000573	0.006761	8.37E-05	0.000302
GO_SIGNAL_TRANSDUCTION_INVOLVED_IN_DNA_DAMAGE_CHECKPOINT	28	Down	0.000573	0.006761	8.37E-05	0.000302
GO_POSITIVE_REGULATION_OF_DNA-DEPENDENT_DNA_REPLICATION	12	Down	0.000574	0.006761	0.000697	0.00072
GO_POSITIVE_REGULATION_OF_POTASSIUM_ION_TRANSMEMBRANE_TRANSPORTER_ACTIVITY	13	Up	0.000575	0.006761	0.000196	0.000306
GO_POSITIVE_REGULATION_OF_I-KAPPAB_KINASE/NF-KAPPAB_SIGNALING	92	Up	0.000576	0.006761	0.000225	0.00032
GO_REGULATION_OF_SENSORY_PERCEPTION_OF_PAIN	26	Up	0.000576	0.006761	0.000104	0.000302
GO_REGULATION_OF_SENSORY_PERCEPTION	26	Up	0.000576	0.006761	0.000104	0.000302
GO_NEGATIVE_REGULATION_OF_TELOMERE_MAINTENANCE_VIA_TELOMERASE	19	Down	0.000576	0.006761	0.000127	0.000302
GO_REGULATION_OF_PHAGOCYTOSIS	77	Up	0.000578	0.006761	0.000196	0.000306
GO_POSITIVE_REGULATION_OF_MYELOID_LEUKOCYTE_MEDIATED_IMMUNITY	29	Up	0.000581	0.006761	0.000242	0.000328
GO_POSITIVE_REGULATION_OF_CELL_CYCLE_G1/S_PHASE_TRANSITION	37	Down	0.000581	0.006761	0.000251	0.000332
GO_REGULATED_EXOCYTOSIS	151	Up	0.000581	0.006761	0.000216	0.000315
GO_NEGATIVE_REGULATION_OF_GLYCOPROTEIN_METABOLIC_PROCESS	14	Up	0.000582	0.006761	1.55E-05	0.000302
GO_DESENSITIZATION_OF_G_PROTEIN-COUPLED_RECEPTOR_SIGNALING_PATHWAY	20	Up	0.000583	0.006761	0.000232	0.000324
GO_NEGATIVE_ADAPTATION_OF_SIGNALING_PATHWAY	20	Up	0.000583	0.006761	0.000232	0.000324
GO_POSITIVE_REGULATION_OF_MACROPHAGE_MIGRATION	17	Up	0.000584	0.006761	9.28E-05	0.000302
GO_NEGATIVE_REGULATION_OF_MICROTUBULE_POLYMERIZATION	12	Up	0.000584	0.006761	5.45E-05	0.000302
GO_REGULATION_OF_MITOTIC_SISTER_CHROMATID_SEPARATION	53	Down	0.000584	0.006761	0.000335	0.000392
GO_MYELOID_LEUKOCYTE_MEDIATED_IMMUNITY	80	Up	0.000584	0.006761	0.000181	0.000302
GO_REGULATION_OF_PROTEIN_HOMODIMERIZATION_ACTIVITY	18	Down	0.000584	0.006761	0.000112	0.000302
GO_CIRCADIAN_SLEEP/WAKE_CYCLE	15	Up	0.000585	0.006761	0.000251	0.000332
GO_PRIMARY_AMINO_COMPOUND_METABOLIC_PROCESS	11	Up	0.000587	0.006761	0.000153	0.000302
GO_CENTRIOLE-CENTRIOLE_COHESION	12	Down	0.000587	0.006761	5.42E-05	0.000302
GO_NEGATIVE_REGULATION_OF_INTRACELLULAR_STEROID_HORMONE_RECEPTOR_SIGNALING_PATHWAY	28	Down	0.000589	0.006761	0.000188	0.000305
GO_RESPONSE_TO_CHOLESTEROL	13	Up	0.000589	0.006761	9.06E-05	0.000302
GO_MAINTENANCE_OF_PROTEIN_LOCALIZATION_IN_ORGANELLE	37	Down	0.00059	0.006761	3.09E-05	0.000302
GO_NEGATIVE_REGULATION_OF_CENTROSOME_CYCLE	13	Down	0.000591	0.006761	0.000298	0.000364
GO_PROTEIN-DNA_COMPLEX_SUBUNIT_ORGANIZATION	151	Down	0.000591	0.006761	0.000283	0.000353
GO_LYMPHOCYTE_APOPTOTIC_PROCESS	76	Up	0.000593	0.006761	0.000107	0.000302
GO_RESPONSE_TO_BACTERIUM	371	Up	0.000593	0.006761	0.000116	0.000302
GO_MONONUCLEAR_CELL_PROLIFERATION	242	Up	0.000594	0.006761	0.000165	0.000302
GO_DOUBLE-STRAND_BREAK_REPAIR_VIA_HOMOLOGOUS_RECOMBINATION	94	Down	0.000594	0.006761	0.000194	0.000306
GO_RECOMBINATIONAL_REPAIR	94	Down	0.000594	0.006761	0.000194	0.000306
GO_CELL_PROJECTION_ASSEMBLY	412	Down	0.000596	0.006761	0.000182	0.000302
GO_POSITIVE_REGULATION_OF_MAST_CELL_ACTIVATION	24	Up	0.000597	0.006761	0.000161	0.000302
GO_REGULATION_OF_CELL_CYCLE_G2/M_PHASE_TRANSITION	87	Down	0.000598	0.006761	0.000229	0.000322

GO_REGULATION_OF_MACROPHAGE_CHEMOTAXIS	24	Up	0.000599	0.006761	9.48E-05	0.000302
GO_REGULATION_OF_IMMUNOGLOBULIN_SECRETION	18	Up	0.000599	0.006761	0.000243	0.000329
GO_POSITIVE_REGULATION_OF_MITOTIC_CELL_CYCLE_PHASE_TRANSITION	63	Down	0.000599	0.006761	0.000256	0.000335
GO_METAPHASE/ANAPHASE_TRANSITION_OF_MITOTIC_CELL_CYCLE	52	Down	0.000601	0.006761	0.00034	0.000395
GO_METAPHASE/ANAPHASE_TRANSITION_OF_CELL_CYCLE	52	Down	0.000601	0.006761	0.00034	0.000395
GO_REGULATION_OF_PEPTIDE_SECRETION	375	Up	0.000603	0.006761	0.000155	0.000302
GO_FLAGELLATED_SPERM_MOTILITY	42	Up	0.000604	0.006761	6.02E-05	0.000302
GO_REPLICATION_FORK_PROCESSING	27	Down	0.000604	0.006761	0.000178	0.000302
GO_KINETOCHORE_ORGANIZATION	19	Down	0.000604	0.006761	0.000317	0.000378
GO_G1/S_TRANSITION_OF_MITOTIC_CELL_CYCLE	162	Down	0.000605	0.006761	0.000195	0.000306
GO_REGULATION_OF_HUMORAL_IMMUNE_RESPONSE	24	Up	0.000609	0.006761	0.000366	0.000416
GO_REGULATION_OF_INTERLEUKIN-6_PRODUCTION	110	Up	0.00061	0.006761	0.000126	0.000302
GO_POLYOL_BIOSYNTHETIC_PROCESS	46	Up	0.000611	0.006761	0.00014	0.000302
GO_RESPONSE_TO_UV	129	Down	0.000611	0.006761	0.000172	0.000302
GO_ORGANOPHOSPHATE_ESTER_TRANSPORT	62	Up	0.000611	0.006761	0.000131	0.000302
GO_POSITIVE_REGULATION_OF_HEMOPOIESIS	174	Up	0.000613	0.006761	0.000108	0.000302
GO_LEUKOCYTE_PROLIFERATION	253	Up	0.000614	0.006761	0.00016	0.000302
GO_REGULATION_OF_B_CELL_DIFFERENTIATION	30	Up	0.000617	0.006761	0.000168	0.000302
GO_FEMALE_MEIOTIC_NUCLEAR_DIVISION	29	Down	0.000617	0.006761	0.000355	0.000407
GO_MITOTIC_SISTER_CHROMATID_SEPARATION	56	Down	0.000617	0.006761	0.000307	0.000371
GO_INTRACELLULAR_ESTROGEN_RECEPTOR_SIGNALING_PATHWAY	39	Down	0.000617	0.006761	0.000372	0.000421
GO_REGULATION_OF_DNA_REPAIR	92	Down	0.000618	0.006761	0.000262	0.000338
GO_G2_DNA_DAMAGE_CHECKPOINT	38	Down	0.000618	0.006761	0.000178	0.000302
GO_LYMPHOCYTE_PROLIFERATION	240	Up	0.00062	0.006761	0.000173	0.000302
GO_GRANULOCYTE_MIGRATION	112	Up	0.000627	0.006761	0.000178	0.000302
GO_REGULATION_OF_G2/M_TRANSITION_OF_MITOTIC_CELL_CYCLE	71	Down	0.000627	0.006761	0.000257	0.000336
GO_PROTEIN_ACTIVATION_CASCADE	38	Up	0.000629	0.006761	0.000241	0.000328
GO_REGULATION_OF_CHROMOSOME_SEPARATION	56	Down	0.000629	0.006761	0.000346	0.000399
GO_MITOTIC_SPINDLE_ORGANIZATION	108	Down	0.00063	0.006761	0.000237	0.000325
GO_REGULATION_OF_MITOTIC_METAPHASE/ANAPHASE_TRANSITION	48	Down	0.00063	0.006761	0.000353	0.000405
GO_REGULATION_OF_METAPHASE/ANAPHASE_TRANSITION_OF_CELL_CYCLE	48	Down	0.00063	0.006761	0.000353	0.000405
GO_DIVALENT_METAL_ION_TRANSPORT	309	Up	0.000631	0.006761	0.000108	0.000302
GO_PEPTIDE_SECRETION	464	Up	0.000632	0.006761	0.000155	0.000302
GO_DNA_LIGATION	13	Down	0.000632	0.006761	0.000239	0.000327
GO_HOMOLOGOUS_CHROMOSOME_SEGREGATION	33	Down	0.000632	0.006761	0.000256	0.000335
GO_DIVALENT_INORGANIC_CATION_TRANSPORT	310	Up	0.000633	0.006761	0.000109	0.000302
GO_CD4-POSITIVE,_ALPHA-BETA_T_CELL_ACTIVATION	77	Up	0.000633	0.006761	0.000147	0.000302
GO_DETECTION_OF_MECHANICAL_STIMULUS_INVOLVED_IN_SENSORY_PERCEPTION_OF_PAIN	11	Up	0.000636	0.006761	0.000141	0.000302
GO_INTERLEUKIN-1-MEDIATED_SIGNALING_PATHWAY	19	Up	0.000636	0.006761	0.000112	0.000302
GO_LYMPH_NODE_DEVELOPMENT	27	Up	0.000637	0.006761	0.000261	0.000337
GO_SECONDARY_METABOLITE_BIOSYNTHETIC_PROCESS	18	Up	0.000639	0.006761	0.000236	0.000325
GO_MEIOTIC_SPINDLE_ORGANIZATION	14	Down	0.00064	0.006761	0.000332	0.000389
GO_REGULATION_OF_OSTEOCLAST_DIFFERENTIATION	60	Up	0.00064	0.006761	0.000165	0.000302
GO_POSITIVE_REGULATION_OF_MYELOID_CELL_DIFFERENTIATION	87	Up	0.000643	0.006761	9.72E-05	0.000302
GO_HETEROTYPIC_CELL-CELL_ADHESION	37	Up	0.000643	0.006761	0.000135	0.000302
GO_POSITIVE_REGULATION_OF_NON-MOTILE_CILIUM_ASSEMBLY	12	Down	0.000644	0.006761	0.000101	0.000302
GO_RESPONSE_TO_LIPOPOLYSACCHARIDE	231	Up	0.000645	0.006761	9.67E-05	0.000302
GO_POSITIVE_REGULATION_OF_IMMUNE_SYSTEM_PROCESS	727	Up	0.000645	0.006761	0.00015	0.000302
GO_NEGATIVE_REGULATION_OF_CELL_CYCLE_PROCESS	199	Down	0.000646	0.006761	0.000153	0.000302
GO_MODIFICATION_BY_SYMBIONT_OF_HOST_MORPHOLOGY_OR_PHYSIOLOGY	27	Up	0.000646	0.006761	6.88E-05	0.000302
GO_CYTOSKELETON-DEPENDENT_CYTOKINESIS	73	Down	0.000646	0.006761	0.000191	0.000306
GO_SPINDLE_ORGANIZATION	157	Down	0.000647	0.006761	0.000227	0.000322
GO_REGULATION_OF_INFLAMMATORY_RESPONSE	246	Up	0.000647	0.006761	0.000134	0.000302
GO_POSITIVE_REGULATION_OF_MONONUCLEAR_CELL_MIGRATION	19	Up	0.000647	0.006761	0.000185	0.000303
GO_PROTEIN_LOCALIZATION_TO_CHROMOSOME,_CENTROMERIC_REGION	22	Down	0.000648	0.006761	0.000387	0.000434
GO_REGULATION_OF_CENTRIOLE_REPLICATION	17	Down	0.000649	0.006761	0.000252	0.000333
GO_CENTROSOME_DUPLICATION	63	Down	0.00065	0.006761	0.000253	0.000333
GO_REGULATION_OF_PH	73	Up	0.00065	0.006761	0.000151	0.000302
GO_CELLULAR_HOMEOSTASIS	613	Up	0.000652	0.006761	0.000128	0.000302
GO_CELLULAR_CHEMICAL_HOMEOSTASIS	518	Up	0.000652	0.006761	0.000121	0.000302
GO_MICROTUBULE_CYTOSKELETON_ORGANIZATION_INVOLVED_IN_MITOSIS	126	Down	0.000653	0.006761	0.000241	0.000328
GO_REGULATION_OF_CELL_CYCLE_CHECKPOINT	32	Down	0.000655	0.006761	0.000154	0.000302
GO_REACTIVE_NITROGEN_SPECIES_METABOLIC_PROCESS	68	Up	0.000655	0.006761	0.000169	0.000302
GO_CALCIUM_ION_TRANSMEMBRANE_TRANSPORT	176	Up	0.000655	0.006761	0.000144	0.000302
GO_POSITIVE_REGULATION_OF_IMMUNE_EFFECTOR_PROCESS	179	Up	0.000656	0.006761	0.000129	0.000302
GO_MYELOID_LEUKOCYTE_MIGRATION	159	Up	0.000657	0.006761	0.000133	0.000302
GO_PROTEIN_K11-LINKED_UBIQUITINATION	24	Down	0.000657	0.006761	0.000159	0.000302
GO_NEGATIVE_REGULATION_OF_LEUKOCYTE_ACTIVATION	145	Up	0.000658	0.006761	0.000134	0.000302
GO_LIPID_STORAGE	53	Up	0.000658	0.006761	0.000228	0.000322
GO_NEGATIVE_REGULATION_OF_CHROMOSOME_SEPARATION	36	Down	0.00066	0.006761	0.000372	0.000421
GO_NEGATIVE_REGULATION_OF_MITOTIC_SISTER_CHROMATID_SEPARATION	36	Down	0.00066	0.006761	0.000372	0.000421
GO_REGULATION_OF_MYELOID_CELL_DIFFERENTIATION	176	Up	0.000661	0.006761	0.000114	0.000302
GO_BONE_RESORPTION	54	Up	0.000667	0.006761	0.000182	0.000302
GO_PH_REDUCTION	35	Up	0.000672	0.006761	0.000142	0.000302
GO_PANCREATIC_JUICE_SECRETION	11	Up	0.000673	0.006761	0.000236	0.000325
GO_REGULATION_OF_LEUKOCYTE_PROLIFERATION	199	Up	0.000674	0.006761	0.000145	0.000302
GO_SEROTONIN_SECRETION	11	Up	0.000674	0.006761	0.000234	0.000324
GO_REGULATION_OF_CYTOKINE_BIOSYNTHETIC_PROCESS	86	Up	0.000676	0.006761	0.000163	0.000302
GO_GRANULOCYTE_CHEMOTAXIS	95	Up	0.000679	0.006761	0.000171	0.000302
GO_CELLULAR_RESPONSE_TO_PH	15	Up	0.000681	0.006761	0.000263	0.000339
GO_DNA_DAMAGE_CHECKPOINT	103	Down	0.000681	0.006761	0.000162	0.000302
GO_INFLAMMATORY_RESPONSE_TO_ANTIGENIC_STIMULUS	42	Up	0.000682	0.006761	0.000146	0.000302
GO_BODY_FLUID_SECRETION	68	Up	0.000684	0.006761	0.000138	0.000302
GO_POSITIVE_REGULATION_OF_ERK1_AND_ERK2_CASCADE	184	Up	0.000684	0.006761	7.76E-05	0.000302
GO_MYELOID_CELL_APOPTOTIC_PROCESS	33	Up	0.000684	0.006761	0.000143	0.000302
GO_REGULATION_OF_LIPID_STORAGE	30	Up	0.000685	0.006761	0.000225	0.00032
GO_NEGATIVE_REGULATION_OF_CENTROSOME_DUPLICATION	12	Down	0.000685	0.006761	0.000319	0.000379
GO_CEREBRAL_CORTEX_RADIAL_GLIA_GUIDED_MIGRATION	18	Down	0.000687	0.006761	0.000231	0.000323
GO_TELENCEPHALON_GLIAL_CELL_MIGRATION	18	Down	0.000687	0.006761	0.000231	0.000323
GO_FEVER_GENERATION	11	Up	0.000687	0.006761	0.000187	0.000304
GO_NEGATIVE_REGULATION_OF_PLATELET-DERIVED_GROWTH_FACTOR_RECEPTOR_SIGNALING_PATHWAY	12	Up	0.000688	0.006761	0.000713	0.000735
GO_POSITIVE_REGULATION_OF_CYTOKINE_SECRETION	118	Up	0.000688	0.006761	0.000198	0.000307
GO_HUMORAL_IMMUNE_RESPONSE	93	Up	0.000689	0.006761	0.000321	0.000381
GO_DNA-DEPENDENT_DNA_REPLICATION_MAINTENANCE_OF_FIDELITY	33	Down	0.000689	0.006761	0.000218	0.000316
GO_NEGATIVE_REGULATION_OF_MITOTIC_METAPHASE/ANAPHASE_TRANSITION	35	Down	0.000691	0.006761	0.000382	0.000429
GO_NEGATIVE_REGULATION_OF_METAPHASE/ANAPHASE_TRANSITION_OF_CELL_CYCLE	35	Down	0.000691	0.006761	0.000382	0.000429
GO_MITOTIC_SPINDLE_CHECKPOINT	34	Down	0.000692	0.006761	0.000374	0.000422
GO_SPINDLE_CHECKPOINT	39	Down	0.000693	0.006761	0.000355	0.000406
GO_NEGATIVE_REGULATION_OF_CELL_ACTIVATION	161	Up	0.000693	0.006761	0.000152	0.000302
GO_POLYOL_METABOLIC_PROCESS	80	Up	0.000694	0.006761	0.00014	0.000302
GO_MITOTIC_SPINDLE_ASSEMBLY_CHECKPOINT	32	Down	0.000696	0.006761	0.000362	0.000412
GO_SPINDLE_ASSEMBLY_CHECKPOINT	32	Down	0.000696	0.006761	0.000362	0.000412
GO_CHRONIC_INFLAMMATORY_RESPONSE	14	Up	0.000697	0.006761	0.00055	0.000579
GO_NEGATIVE_REGULATION_OF_SECRETION_BY_CELL	155	Up	0.000697	0.006761	0.000129	0.000302
GO_SECONDARY_METABOLIC_PROCESS	49	Up	0.000698	0.006761	0.000226	0.000321
GO_SEQUESTERING_OF_CALCIUM_ION	82	Up	0.000699	0.006761	0.000134	0.000302
GO_REGULATION_OF_SEQUESTERING_OF_CALCIUM_ION	82	Up	0.000699	0.006761	0.000134	0.000302
GO_INTERLEUKIN-12_PRODUCTION	51	Up	0.000699	0.006761	0.000216	0.000315
GO_IMMUNOLOGICAL_SYNAPSE_FORMATION	12	Up	0.000701	0.006761	0.000437	0.000478
GO_DNA_REPLICATION-INDEPENDENT_NUCLEOSOME_ASSEMBLY	16	Down	0.000702	0.006761	0.00041	0.000453
GO_REGULATION_OF_T_CELL_APOPTOTIC_PROCESS	40	Up	0.000702	0.006761	8.15E-05	0.000302
GO_REGULATION_OF_MONONUCLEAR_CELL_PROLIFERATION	193	Up	0.000702	0.006761	0.000145	0.000302
GO_POSITIVE_REGULATION_OF_OXIDOREDUCTASE_ACTIVITY	38	Up	0.000703	0.006761	0.000194	0.000306
GO_CHROMOSOME_SEPARATION	78	Down	0.000708	0.006761	0.000311	0.000374
GO_NUCLEOSOME_ASSEMBLY	58	Down	0.000709	0.006761	0.000459	0.000497
GO_REGULATION_OF_DOUBLE-STRAND_BREAK_REPAIR_VIA_HOMOLOGOUS_RECOMBINATION	28	Down	0.000709	0.006761	0.000294	0.000362
GO_REGULATION_OF_RESPONSE_TO_DNA_DAMAGE_STIMULUS	176	Down	0.000712	0.006761	0.000181	0.000302
GO_RELEASE_OF_SEQUESTERED_CALCIUM_ION_INTO_CYTOSOL	81	Up	0.000713	0.006761	0.000121	0.000302
GO_NEGATIVE_REGULATION_OF_SEQUESTERING_OF_CALCIUM_ION	81	Up	0.000713	0.006761	0.000121	0.000302
GO_REGULATION_OF_TUMOR_NECROSIS_FACTOR-MEDIATED_SIGNALING_PATHWAY	23	Up	0.000714	0.006761	0.000264	0.00034

Supplementary Table 2. List of antibodies used in western blotting, immunohistochemistry, and flow cytometry. All flow cytometry antibodies were rat anti-mouse.

Target	Clone(s)	Fluorochrome	Dilution	Source
PDCD4	D29C6	N/A: For western	1:1,000	CST
PTEN	D4.3	N/A: For western	1:1,000	CST
HSP90	EPR16621-67	N/A: For western	1:1,000	Abcam
CD45	30-F11	N/A: For IHC	1:200	BD Pharmingen
Cyclin D1	E3P5S	NA: For western and IHC	WB: 1:1,500<br>IHC: 1:500	CST
Phospho-histone H3 (S10)	Cat#9701	NA: For western and IHC	WB: 1:1,500<br>IHC: 1:500	CST
Cleaved caspase 3 (D175)	Cat#9661	NA: For western and IHC	WB: 1:1,500<br>IHC: 1:500	CST
BAX	Cat#554106	NA: For western and IHC	WB: 1:1,500<br>IHC: 1:500	BD Pharmingen
CD4	4SM95	N/A: For IHC	1:200	Invitrogen
CD8α	4SM15	N/A: For IHC	1:200	eBioscience/Affymetrix
CD16/CD32	2.4G2	N/A	1:300	BD Biosciences
CD45.2	104	APC	1:300	eBioscience/Invitrogen
TCRβ	H57-597	PE-Cy7	1:200	BD Biosciences
CD8α	53-6.7	APC-Cy7	1:400	BD Biosciences
CD4	RM4.5	PE-Cy5	1:500	BD Biosciences
CD69	H1.2F3	PE	1:100	eBioscience/Invitrogen
CD49b	Dx5	BV421	1:100	BD Biosciences
CD11b	M1/70	APC-Cy7	1:1,000	BD Biosciences
Ly6C	AL-21	BV421	1:500	BD Biosciences
Ly6G	1A8	PE-Cy7	1:1,000	BD Biosciences
CD206	C068C2	BV711	1:200	BioLegend
PD-L1	MIH5	PE	1:200	eBioscience/Invitrogen
CD11c	HL3	BV510	1:100	BD Biosciences
MHC class II	M5/114.15.2	PE	1:400	eBioscience/Invitrogen
NKp46	29A1.4	BV510	1:100	BD Biosciences

Figure S1

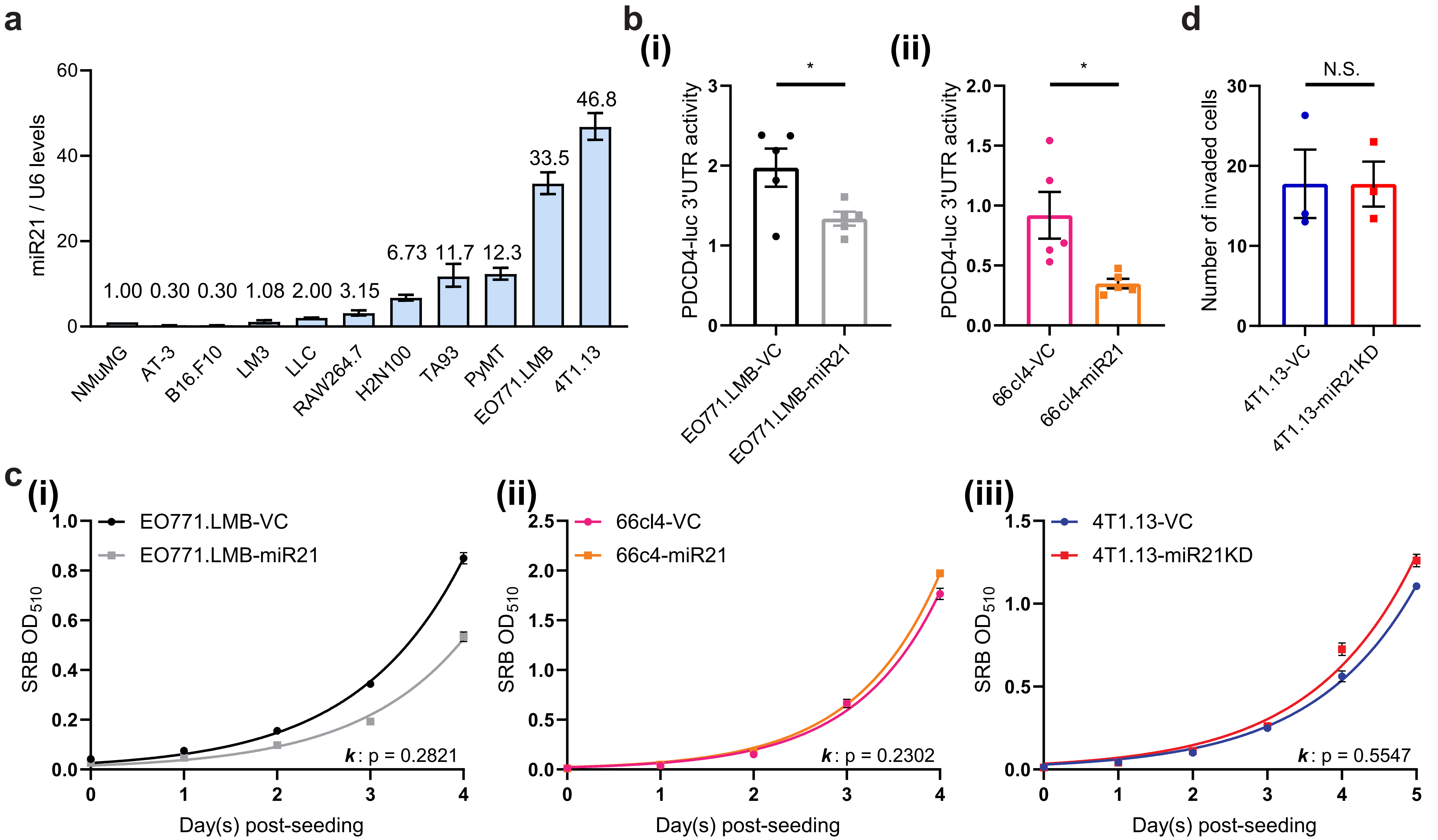
a
miR21 / U6 levels
1.00 0.30 0.30 1.08 2.00 3.15 6.73 11.7 12.3 33.5 46.8
NMuMG AT-3 B16.F10 LM3 LLC RAW264.7 H2N100 TA93 PyMT EO771.LMB 4T1.13
b
(i)
PDCD4-luc 3'UTR activity
*
EO771.LMB-VC EO771.LMB-miR21
(ii)
PDCD4-luc 3'UTR activity
*
66cl4-VC 66cl4-miR21
d
Number of invaded cells
N.S.
4T1.13-VC 4T1.13-miR21KD
c
(i)
EO771.LMB-VC
EO771.LMB-miR21
SRB OD510
k: p = 0.2821
Day(s) post-seeding
(ii)
66cl4-VC
66c4-miR21
SRB OD510
k: p = 0.2302
Day(s) post-seeding
(iii)
4T1.13-VC
4T1.13-miR21KD
SRB OD510
k: p = 0.5547
Day(s) post-seeding

Figure S1. Effect of miR-21 on mouse mammary cancer cells *in vitro*.

(a) MiR-21 expression levels in a panel of normal or transformed mouse cell lines. Mean +/- SEM (n=3). Expression in NMuMG immortalised mouse mammary epithelial cells was set to 1. **(b)** Validation of miR-21 overexpression in EO771.LMB (i) and 66cl4 (ii) cells as measured by PDCD4 3'UTR reporter gene activity. Mean +/- SEM (n=5). **(c)** Effect of modulation of miR-21 levels or activity on the proliferative capacity of EO771.LMB (i), 66cl4 (ii) and 4T1.13 cells (iii). Mean +/- SEM (n=6). Statistical analysis of the proliferation rate parameter (k) was performed by the exponential growth equation function in Prism. **(d)** Effect of loss of miR-21 activity on the invasive capacity of 4T1.13 cells. Mean +/- SEM (n=3).

Figure S2

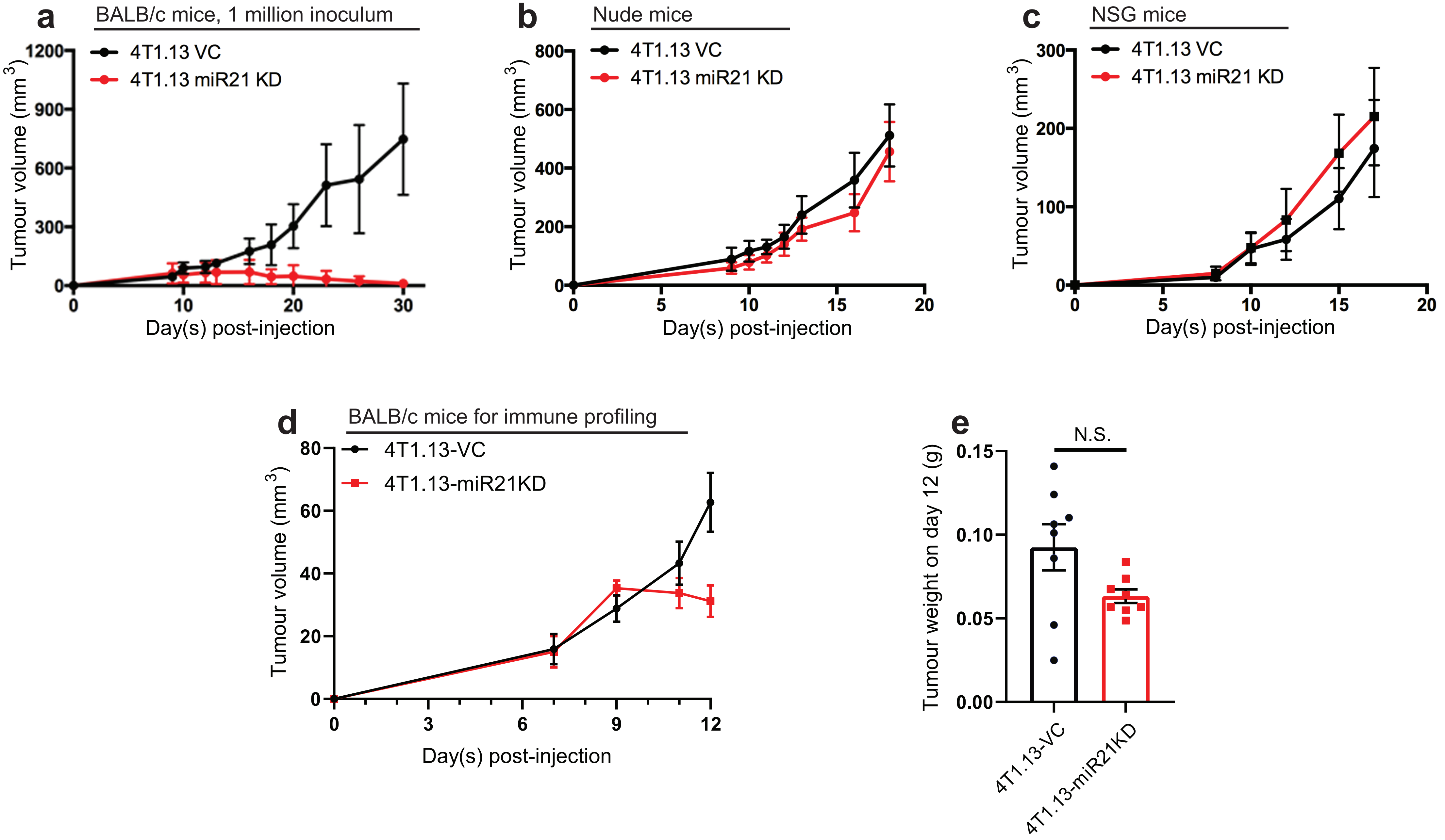

a
BALB/c mice, 1 million inoculum
4T1.13 VC
4T1.13 miR21 KD
Tumour volume (mm^3)
0
300
600
900
1200
0
10
20
30
Day(s) post-injection
b
Nude mice
4T1.13 VC
4T1.13 miR21 KD
Tumour volume (mm^3)
0
200
400
600
800
0
5
10
15
20
Day(s) post-injection
c
NSG mice
4T1.13 VC
4T1.13 miR21 KD
Tumour volume (mm^3)
0
100
200
300
0
5
10
15
20
Day(s) post-injection
d
BALB/c mice for immune profiling
4T1.13-VC
4T1.13-miR21KD
Tumour volume (mm^3)
0
20
40
60
80
0
3
6
9
12
Day(s) post-injection
e
N.S.
Tumour weight on day 12 (g)
0.00
0.05
0.10
0.15
4T1.13-VC
4T1.13-miR21KD

Figure S2. Effect of miR-21 on mammary tumour growth.

a) Effect of loss of miR-21 activity on the growth rate of 4T1.13 mammary tumours, following an increased inoculum of $1x10^6$ cells (n=13). **(b-c)** Effect of loss of miR-21 activity on the growth rate of 4T1.13 tumours in Balb/c nu/nu **(b)** or NSG **(c)** mice following an inoculum of $2x10^5$ cells (n=10). **(d-e)** Growth kinetics and weights of 4T1.13 tumours on day 12 in BALB/c mice used for immune profiling in Figure 4. Displayed as mean+/-SEM.

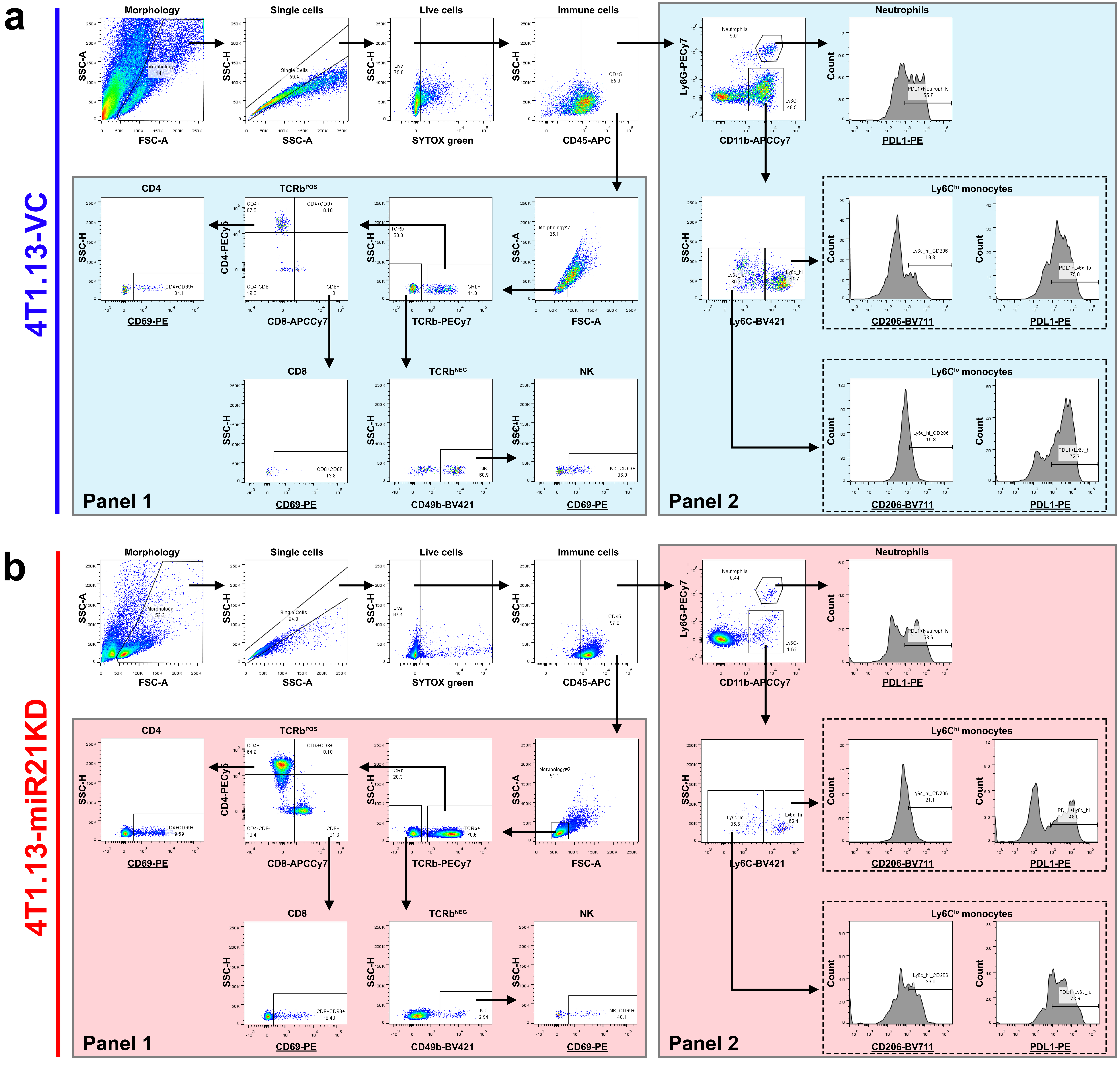
a
4T1.13-VC
Morphology
Single cells
Live cells
Immune cells
Neutrophils
FSC-A
SSC-A
SSC-H
SYTOX green
CD45-APC
CD11b-APCCy7
Ly6G-PECy7
PDL1-PE
Count
CD4
TCRb^POS
CD69-PE
CD8-APCCy7
CD4-PECy5
TCRb-PECy7
Ly6C-BV421
Ly6C^hi monocytes
CD206-BV711
Ly6C^lo monocytes
CD8
TCRb^NEG
NK
CD49b-BV421
Panel 1
Panel 2
b
4T1.13-miR21KD

Figure S3. Gating strategies for lymphocytes and myeloid cells in 4T1.13 tumours. The antibodies used are listed in **Table S2**. Gating for underlined antibodies was based on the fluorescence minus one (FMO) controls.

Figure S4

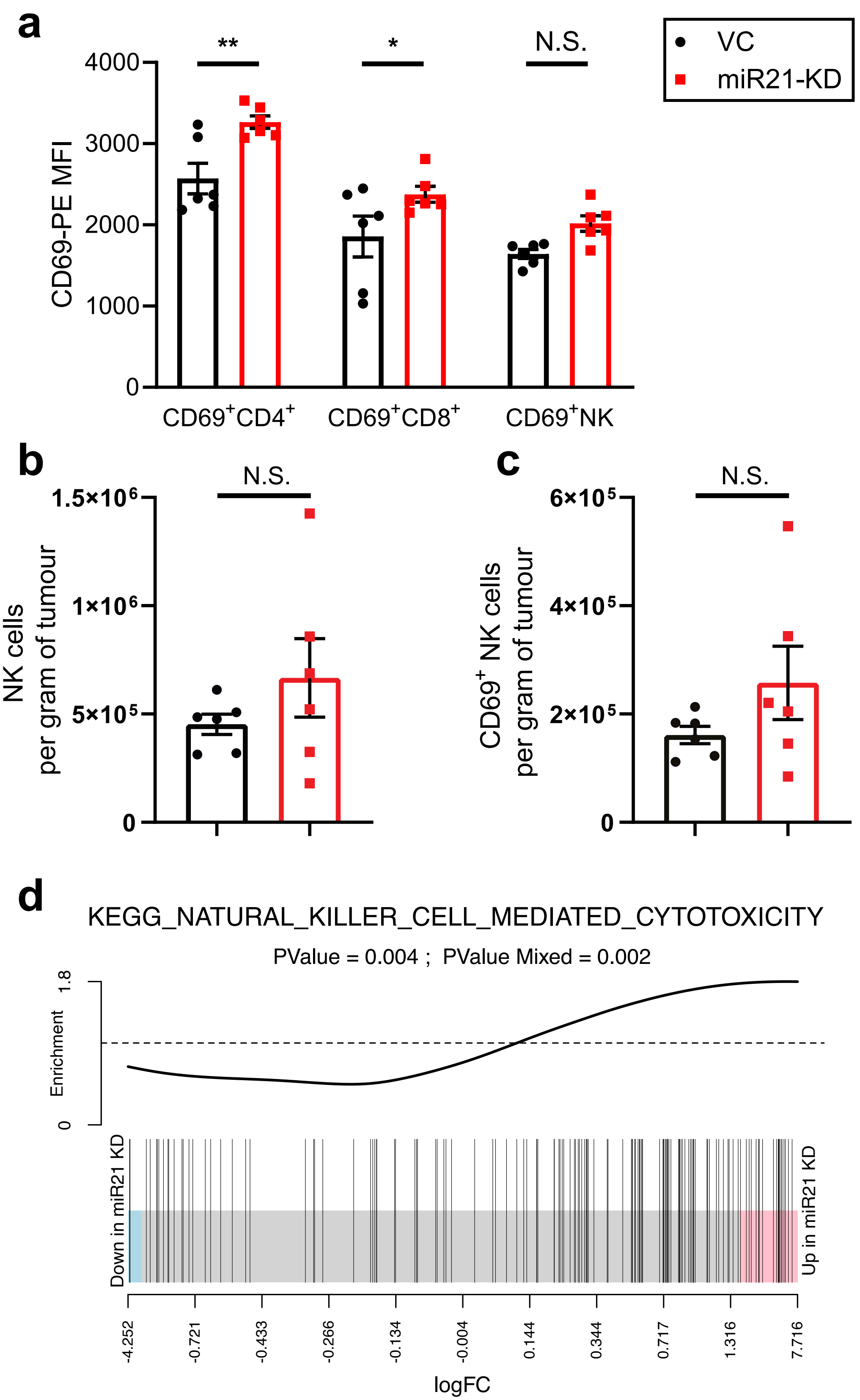

a
**
*
N.S.
VC
miR21-KD
CD69-PE MFI
4000
3000
2000
1000
0
CD69+CD4+
CD69+CD8+
CD69+NK
b
N.S.
NK cells per gram of tumour
1.5×10⁶
1×10⁶
5×10⁵
0
c
N.S.
CD69+ NK cells per gram of tumour
6×10⁵
4×10⁵
2×10⁵
0
d
KEGG_NATURAL_KILLER_CELL_MEDIATED_CYTOTOXICITY
PValue = 0.004 ; PValue Mixed = 0.002
Enrichment
1.8
0
Down in miR21 KD
Up in miR21 KD
-4.252
-0.721
-0.433
-0.266
-0.134
-0.004
0.144
0.344
0.717
1.316
7.716
logFC

Figure S4. Immune profiling of 4T1.13 tumours

(**a**) Mean fluorescence intensity (MFI) of CD69 on $CD4^+$ and $CD8^+$T cells and on NK cells infiltrating 4T1.13-VC and 4T1.13-miR-21-KD tumours. **(b-c)** Effect of loss of miR-21 activity on the number of NK cells (b) or $CD69^+$NK cells (c) infiltrating 4T1.13-VC and 4T1.13-miR-21-KD tumours. (**d**) Barcode plot of enrichment of NK cell mediated cytotoxicity in the 4T1.13-miR-21-KD tumours.

Figure S5

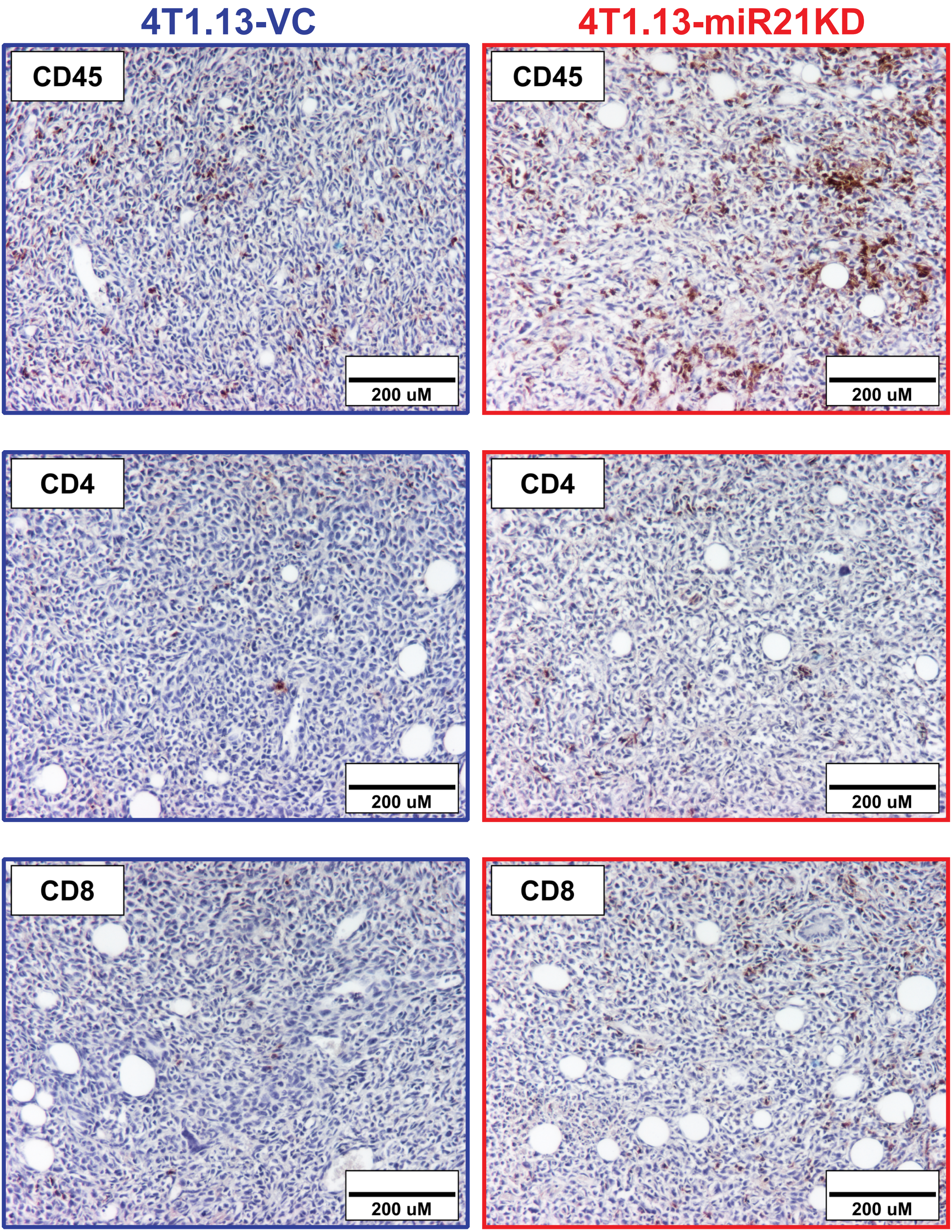
4T1.13-VC
4T1.13-miR21KD
CD45
CD45
200 uM
200 uM
CD4
CD4
200 uM
200 uM
CD8
CD8
200 uM
200 uM

Figure S5. Immunohistochemical validation of T cells.

Changes in $CD45^+$ total immune cell infiltrates, in $CD4^+$ and $CD8^+$T cell infiltrates in 4T1.13-miR-21-KD compared to 4T1.13-VC tumours as assessed by immunohistochemistry (VC: EO771.LMB-VC; OE: EO771.LMB-miR-21).

Figure S6

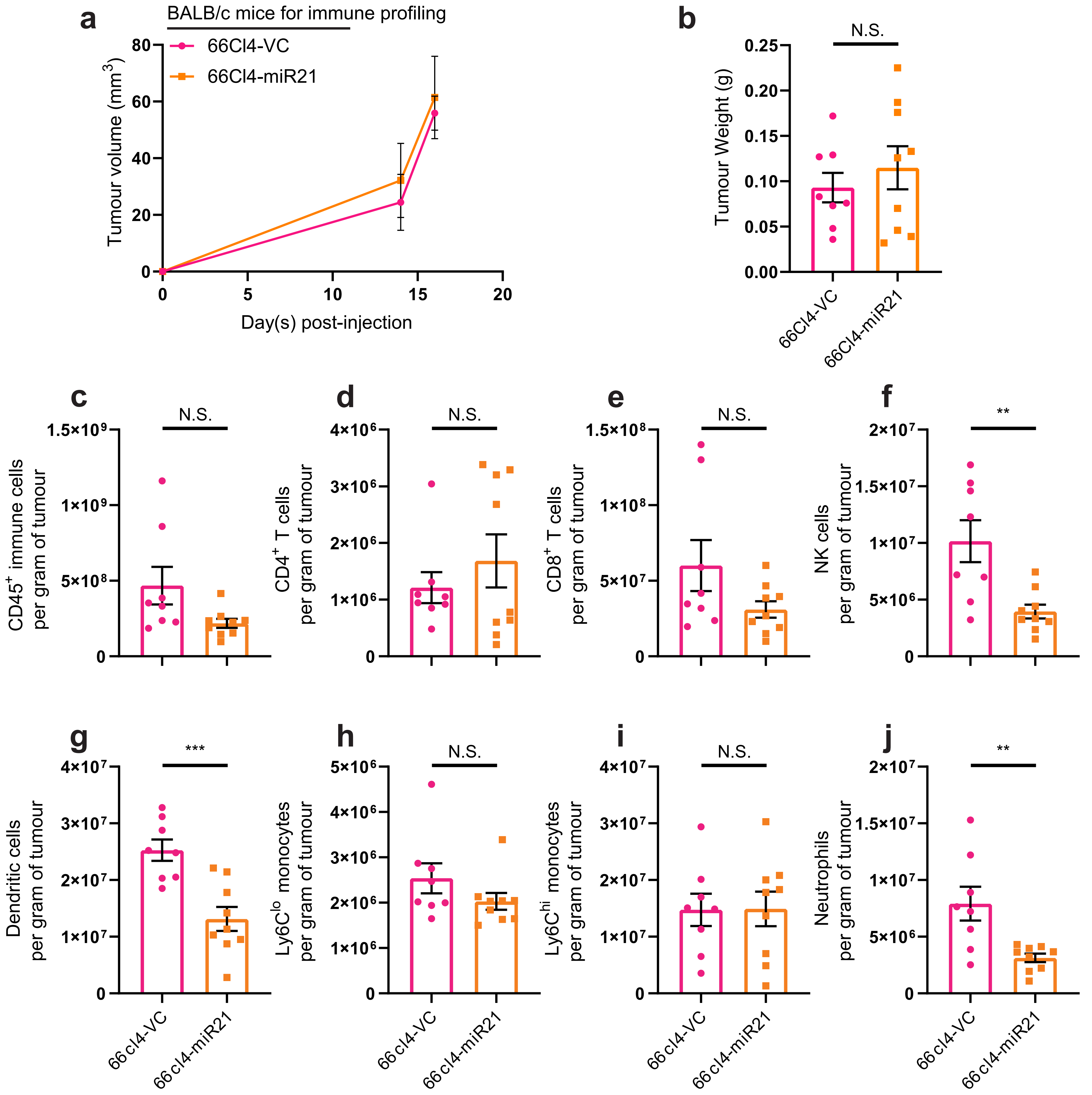
a
BALB/c mice for immune profiling
66Cl4-VC
66Cl4-miR21
Tumour volume (mm^3)
Day(s) post-injection
b
N.S.
Tumour Weight (g)
66Cl4-VC
66Cl4-miR21
c
N.S.
CD45$^+$ immune cells per gram of tumour
d
N.S.
CD4$^+$ T cells per gram of tumour
e
N.S.
CD8$^+$ T cells per gram of tumour
f
**
NK cells per gram of tumour
g
***
Dendritic cells per gram of tumour
h
N.S.
Ly6C^{lo} monocytes per gram of tumour
i
N.S.
Ly6C^{hi} monocytes per gram of tumour
j
**
Neutrophils per gram of tumour
66cl4-VC
66cl4-miR21

Figure S6. Immune profiling of 66cl4 tumours with miR-21 overexpression.

(a-b) Growth kinetics and weights of 66cl4 tumours used for immune profiling. Cells ($5x10^5$) were injected into the mammary gland of BALB/c mice and removed on day 17 for immune cell profiling. Mean +/- SEM (n=8 for the 66cl4-VC group, and n=9 for the 66cl4-miR-21 group). **(c-j)** Total numbers of immune infiltrates per gram of tumour in the 66cl4-VC and 66cl4-miR-21 groups. Mean +/- SEM (n=8 for the 66cl4-VC group, and n=9 for the 66cl4-miR-21 group).

Figure S7

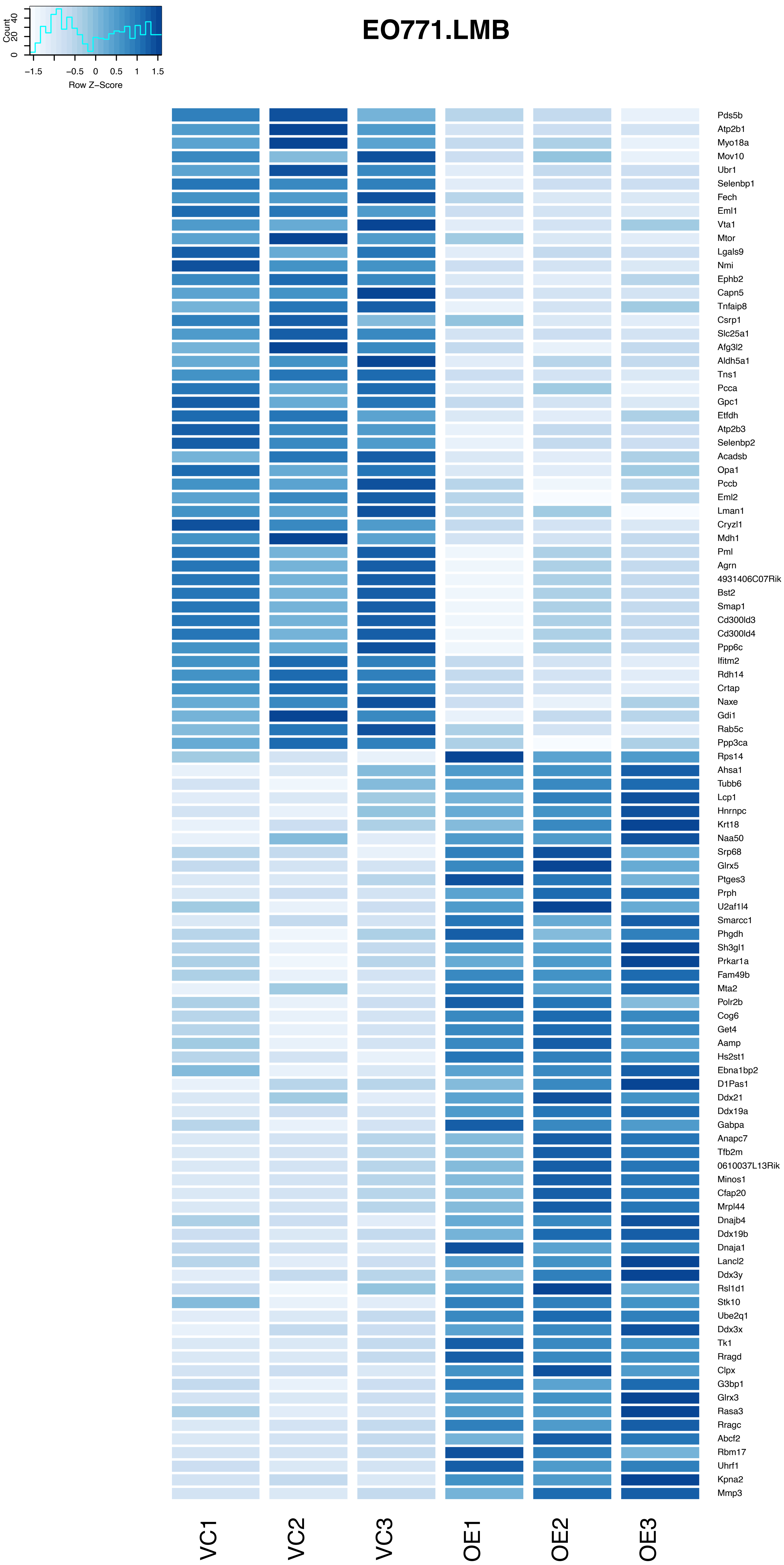

Count
0 20 40
−1.5 −0.5 0 0.5 1 1.5
Row Z−Score
EO771.LMB
Pds5b
Atp2b1
Myo18a
Mov10
Ubr1
Selenbp1
Fech
Eml1
Vta1
Mtor
Lgals9
Nmi
Ephb2
Capn5
Tnfaip8
Csrp1
Slc25a1
Afg3l2
Aldh5a1
Tns1
Pcca
Gpc1
Etfdh
Atp2b3
Selenbp2
Acadsb
Opa1
Pccb
Eml2
Lman1
Cryzl1
Mdh1
Pml
Agrn
4931406C07Rik
Bst2
Smap1
Cd300ld3
Cd300ld4
Ppp6c
Ifitm2
Rdh14
Crtap
Naxe
Gdi1
Rab5c
Ppp3ca
Rps14
Ahsa1
Tubb6
Lcp1
Hnrnpc
Krt18
Naa50
Srp68
Glrx5
Ptges3
Prph
U2af1l4
Smarcc1
Phgdh
Sh3gl1
Prkar1a
Fam49b
Mta2
Polr2b
Cog6
Get4
Aamp
Hs2st1
Ebna1bp2
D1Pas1
Ddx21
Ddx19a
Gabpa
Anapc7
Tfb2m
0610037L13Rik
Minos1
Cfap20
Mrpl44
Dnajb4
Ddx19b
Dnaja1
Lancl2
Ddx3y
Rsl1d1
Stk10
Ube2q1
Ddx3x
Tk1
Rragd
Clpx
G3bp1
Glrx3
Rasa3
Rragc
Abcf2
Rbm17
Uhrf1
Kpna2
Mmp3
VC1
VC2
VC3
OE1
OE2
OE3

Figure S7. Proteomic analysis of EO771.LMB-VC and EO771.LMB-miR-21 cells, showing the top differentially expressed proteins in triplicate samples of the two cell lines.
